# Intermobility of barium, strontium, and lead in chloride and sulfate leach solutions

**DOI:** 10.1186/s12932-019-0064-0

**Published:** 2019-09-05

**Authors:** Mark Rollog, Nigel J. Cook, Paul Guagliardo, Kathy Ehrig, Sarah E. Gilbert, Matt Kilburn

**Affiliations:** 1 School of Chemical Engineering, The University of Adelaide, Adelaide, SA 5005 Australia; 2 Centre for Microscopy, Characterisation, and Analysis, The University of Western Australia, 35 Stirling Highway, Crawley, WA 6009 Australia; 3 BHP Olympic Dam, 55 Grenfell St., Adelaide, SA 5000 Australia; 4 Adelaide Microscopy, The University of Adelaide, Adelaide, SA 5005 Australia

**Keywords:** Alkali earth sulfates, Sulfate leaching, NanoSIMS analysis, Cation intermobility, Radionuclides

## Abstract

Production of radionuclide-free copper concentrates is dependent on understanding and controlling the deportment of daughter radionuclides (RNs) produced from ^238^U decay, specifically ^226^Ra, ^210^Pb, and ^210^Po. Sulfuric acid leaching is currently employed in the Olympic Dam processing plant (South Australia) to remove U and fluorine from copper concentrates prior to smelting but does not adequately remove the aforementioned RN. Due to chemical similarities between lead and alkaline earth metals (including Ra), two sets of experiments were designed to understand solution interactions between Sr, Ba, and Pb at various conditions. Nanoscale secondary ion mass spectrometry (NanoSIMS) isotopic spatial distribution maps and laser ablation inductively coupled-plasma mass spectrometry transects were performed on laboratory-grown crystals of baryte, celestite, and anglesite which had been exposed to different solutions under different pH and reaction time conditions. Analysis of experimental products reveals three uptake mechanisms: overgrowth of nearly pure SrSO_4_ and PbSO_4_ on baryte; incorporation of minor of Pb and Ba into celestite due to diffusion; and extensive replacement of Pb by Sr (and less extensive replacement of Pb by Ba) in anglesite via coupled dissolution-reprecipitation reactions. The presence of H_2_SO_4_ either enhanced or inhibited these reactions. Kinetic modelling supports the experimental results, showing potential for extrapolating the (Sr, Ba, Pb)SO_4_ system to encompass RaSO_4_. Direct observation of grain-scale element distributions by nanoSIMS aids understanding of the controlling conditions and mechanisms of replacement that may be critical steps for Pb and Ra removal from concentrates by allowing construction of a cationic replacement scenario targeting Pb or Ra, or ideally all insoluble sulfates. Experimental results provide a foundation for further investigation of RN uptake during minerals processing, especially during acid leaching. The new evidence enhances understanding of micro- to nanoscale chemical interactions and not only aids determination of where radionuclides reside during each processing stage but also guides development of flowsheets targeting their removal.

## Introduction

Uranium-bearing mineral deposits, such as the Olympic Dam iron oxide-copper-gold-uranium (IOCG-U) orebody, South Australia, contain not only appreciable amounts of uranium and thorium, but also all daughter isotopes produced by radioactive decay. Elimination or reduction of some daughter radionuclides (RNs) during processing represents a unique metallurgical challenge. As examples, ^226^Ra, ^210^Pb, and ^210^Po are all found in Olympic Dam ore feed at sub-ppb concentrations. To achieve activities of < 1 Bq/g per radionuclide in the final copper concentrate, concentrations of roughly 27 parts-per-trillion (ppt), 370 parts-per-quadrillion (ppq), and 6 ppq, respectively, are required. Since these concentration values fall below the minimum detection limits of most conventional instrumentation for analysis of samples in situ, it is simpler to use proxies, where possible, to predict the behavior of these elements during processing.

Understanding hydrothermal mobility of cations in ore deposits is important for the study of ore genesis, but this information may be of limited usefulness on the processing floor. Hydrometallurgy involving high-temperature leach solutions containing acids, alkalis, complexing agents, organic solvents, redox reagents, or more likely a combination of the above, can completely rearrange the chemical composition of ore material—preferentially to the operator’s benefit. Sulfuric acid leaching is a key solution currently employed in the Olympic Dam plant to reduce U and RNs in copper concentrates prior to smelting (8 to 12-h leach time at ~ 60 °C and pH of ~ 1–1.5). A simplified Olympic Dam processing flowsheet is presented in Schmandt et al. [38]. Since the chlorides and nitrates of Ra, Po, and to some extent Pb, are all water- and acid-soluble, these are not of primary concern. Sulfates of these cations, however, are of great interest due to their insolubility and potential for radionuclide sequestration.

Before attempting to determine the deportment of Ra, Po or ^210^Pb, it is vital to understand intermobility of Ba, Sr and Pb among their insoluble sulfates—the minerals baryte, celestite, and anglesite. A significant amount of work has been done in this field, primarily on individual compounds. Strontium sulfate solubility in water [16, 20, 32], in chloride solutions [5, 20, 32], and in sulfate solutions [20] has been determined, as has extraction and biosorption of Sr in the environment [14, 43]. Barium sulfate solubility in water [16, 31], chloride solutions [4, 5, 7, 31], and sulfate solutions [7] has likewise been covered, notably from researchers interested in boiler scale. Lead sulfate solubility greatly affects lead-acid battery performance and has therefore been extensively measured in water [18], and in chloride [21] and sulfate solutions [17, 18, 21]. Radium solubilities have also been determined [3, 44]. These are but a few of the studies addressing solubilities in the entire Sr-Ba-Pb-Ra-Cl-SO_4_-H^+^-H_2_O system. Current consensus is that solubility of the alkali metal (and lead) sulfates in water at 60 °C is Mg > Ca > Sr > Pb > Ba > Ra. Generally, solubilities positively correlate with chloride activity (through complexation), although SrSO_4_ solubility reaches a maximum between 2 and 3 N NaCl or HCl, decreasing at higher concentrations [20]. This would suggest a simple chloride leach may be a potential approach to removal of selected RNs, but the system is far more complex than it initially appears.

Process waters at Olympic Dam contain chloride (e.g., flotation water is 2.5 to 4 g/L Cl^−^), as does the ore itself, but the sulfuric acid leach process (involving up to 150 g/L sulfate) overwhelmingly dictates solution activity and pH. From a RN standpoint, sulfuric acid would be the least favorable reactant due to the insolubility of RaSO_4_, PbSO_4_, and PoSO_4_. Nonetheless, efficiency in removal of fluorine as well as dissolution of most uranium/thorium and rare earth species—coupled with low cost—makes sulfuric acid the logical, practical choice. To that end, optimizing the process already in place is preferential to redesigning the entire system. With additional information about nanoscale mineral-fluid reactions and the behavior of RN-sulfate nanoparticles, it may be possible to modify existing industrial processes to minimize their accumulation in economic products.

To elicit this information, two methods were employed. Laser ablation inductively coupled-plasma mass spectrometry (LA-ICP-MS) is a powerful, well-established tool for generating quantitative compositional data in solids, and is accordingly widely applied across the earth sciences and in mineral processing research [6]. It has, however, several drawbacks. Quadrupole mass spectrometry generally has a mass resolution of 1 atomic mass unit (amu), which prevents distinction between the mass of interest and isobaric mass interferences. Additionally, the finest spatial resolution available is limited by a minimum 3 μm-diameter spot (commonly resulting in a much larger pit, depending on the mineral). For quantitative trace element analysis, much larger spot sizes are required. The Cameca nanoscale secondary ion mass spectrometry (nanoSIMS) platform is an imaging technique which offers solutions to both the above problems. Each of seven detectors on the nanoSIMS has mass resolution approaching 0.1 amu, which is very useful in distinguishing, for example, ^226^Ra (226.0254) from ^88^Sr^138^Ba (225.811). Additionally, the effective spot size for high concentration elements can be < 100 nm, although for trace elements may approach 700 nm. This still represents a significant improvement over LA-ICP-MS for the resolution of nanoscale features. NanoSIMS, however, is not currently quantifiable - at least not for mineral analyses. Although each is independently limited, the complementary use of both methods provides the quantification and spatial resolution necessary for the results required in this investigation.

Through the combined analyses provided by these two analytical platforms, we strive to better understand the deportment of Sr, Ba, Pb, and by extension, also Ra, throughout ore processing at Olympic Dam. Process methods are ever-changing; optimization is achieved via complex formulae based on mineral abundances, elemental compositions, operating costs, and time—weighed against the constantly moving targets of commodity prices. Even slight adjustments in certain mathematical expressions may result in significant benefit to the operators, so it is crucial to understand (to the extent possible and/or realistically implementable) the intimate mechanisms responsible for the behavior of selected elements—either beneficial or detrimental—during processing. Beyond minerals processing, these results provide valuable insight regarding mechanisms involved in natural processes such as ore formation, hydrothermal alteration, and weathering—and anthropogenic processes including soil reclamation, boiler scale prevention, and nuclear waste storage.

## Experimental methods

### Raw material synthesis and characterization

#### Crystal growth

To control purity, synthetic mineral crystals were produced using a gel-growth method [13, 22]. A ~ 0.5 M sodium metasilicate stock solution was prepared by adding 100 g Na_2_SiO_3_·5H_2_O to 1 L of reverse osmosis (RO) water (boiled and cooled to remove CO_2_). One drop of bromophenol blue indicator was added to 20 mL of stock solution, with stirring, and 3 M HCl was added in small portions until the loss of blue color indicated a pH of < 4.5. SrCl_2_, BaCl_2_ or Pb(acetate)_2_ solution (0.5 mL, 1 M) was added dropwise, with stirring. The solutions were quickly poured into glass test tubes, 2.5 cm in diameter and 15 cm long, lightly covered, and allowed to set for 1 week. A K_2_SO_4_ solution (10 mL, 1 M) was added slowly to the top of the semi-firm gels, taking care not to disrupt the surface. Crystals grew by diffusion within 1–2 weeks and were well-formed, ranging from < 100 μm to > 500 μm in length.

#### Characterization methods

Samples from each batch were analyzed using a FEI Quanta 450 field emission gun scanning electron microscope (FEG-SEM) equipped with an EDAX energy- dispersive X-ray (EDS) detector (Adelaide Microscopy, The University of Adelaide) to verify composition and quality.

### Leaching/recrystallization tests

#### Reactions in simple solutions

To elicit information regarding the uptake of competing cations, crystals were exposed to solutions of single cations under various anionic activity and time conditions. Table 1 lists the contents and conditions of the 24 vials. Half of these were run with only MCl_2_ solution (M = Sr, Ba, or Pb) while the other half also included 1.6 M H_2_SO_4_ to more closely represent the conditions found in a typical acid leach tank. As expected, white sulfate precipitated immediately in all reaction experiments containing sulfuric acid, resulting in reduced effective concentrations of all three cation solutions. The reduced concentrations should reasonably reproduce actual activities present during processing in a 1.6 M H_2_SO_4_ acid leach solution.

**Table 1:** Experimental conditions for reactions in simple solutions

Experiment/vial	Crystals	a. 40 h	b. 40 h	c. 210 h	d. 210 h
5(a–d)	BaSO_4_	0.07 M PbCl_2_	0.07 M PbCl_2_* 1.6 M H_2_SO_4_	0.07 M PbCl_2_	0.07 M PbCl_2_* 1.6 M H_2_SO_4_
6(a–d)	BaSO_4_	0.1 M SrCl_2_	0.1 M SrCl_2_* 1.6 M H_2_SO_4_	0.1 M SrCl_2_	0.1 M SrCl_2_* 1.6 M H_2_SO_4_
7(a–d)	SrSO_4_	0.1 M BaCl_2_	0.1 M BaCl_2_* 1.6 M H_2_SO_4_	0.1 M BaCl_2_	0.1 M BaCl_2_* 1.6 M H_2_SO_4_
8(a–d)	SrSO_4_	0.07 M PbCl_2_	0.07 M PbCl_2_* 1.6 M H_2_SO_4_	0.07 M PbCl_2_	0.07 M PbCl_2_* 1.6 M H_2_SO_4_
9(a–d)	PbSO_4_	0.1 M SrCl_2_	0.1 M SrCl_2_* 1.6 M H_2_SO_4_	0.1 M SrCl_2_	0.1 M SrCl_2_* 1.6 M H_2_SO_4_
10(a–d)	PbSO_4_	0.1 M BaCl_2_	0.1 M BaCl_2_* 1.6 M H_2_SO_4_	0.1 M BaCl_2_	0.1 M BaCl_2_* 1.6 M H_2_SO_4_

*Estimated

Vials were capped and heated to 60 °C (typical for Olympic Dam hydrometallurgical processes) for either 40 or 210 h. Without cooling, the surviving crystals were rinsed with 60 °C RO water three times, dried, individually selected and embedded in 2.5 cm-round epoxy resin mounts. The mounts were polished, carbon-coated, and imaged in backscatter electron (BSE) mode by SEM. The primary distinction between this experiment and the one below is that these crystals were isolated and only exposed to one additional cation at a time, in great excess.

#### Crystal analysis by LA-ICP-MS

Samples from the above set of experiments were analyzed by LA-ICP-MS using an ASI RESOlution-LR ArF excimer laser ablation system equipped with a large format S155 sample chamber (Laurin Technic Inc.) and coupled to an Agilent 7900 × ICP-MS. Transects were performed across each crystal, including at least an extra 10 μm on either side in the epoxy to establish a blank. Instrument conditions for the transects were set using a 6 μm spot size, fluence 3.5 J/cm^2^, repetition rate 10 Hz. The NIST-610 reference standard was analyzed in replicate at the beginning, middle, and end of the run, with two sections of 24 transects in-between. Standards were run using a 74 μm spot size, fluence 3.5 J/cm^2^, repetition rate 10 Hz. Isotopes analyzed were limited to ^35^Cl, ^88^Sr, ^138^Ba, ^204, 206, 207, 208^Pb, and ^226^Ra. Due to the simple, stoichiometric composition of the crystals, elemental concentration data (in ppm) was calculated using a modified version of the internal standard method [19] with an additional minor drift correction. To avoid irregularities at grain edges, concentration values were calculated in ppm normalized to 1,000,000 instead of ppm_(total count)_. Isotopic concentrations were converted to elemental concentrations using global isotopic abundances [12]. The time-resolved transect data from the ICP-MS (in seconds) was converted to distance (μm) by direct comparison between transect traces and their corresponding BSE image, and are therefore estimates. Calculated concentration data was smoothed using a 3-period moving average to minimize electronic spikes.

Despite clean EDS spectra, LA-ICP-MS analyses revealed that the crystals were slightly contaminated with other cations. As a result, the baryte crystals contained approximately 10 ppm Pb and 140 ppm Sr; the celestite contained approximately 10 ppm each of Ba and Pb; and the anglesite contained around 165 ppm Ba and 115 ppm Sr. The SrCl_2_, BaCl_2_, and PbCl_2_ solutions also contained ppm quantities of contaminants, but analysis of the data suggests that contamination of both crystals and solutions proved to be many orders of magnitude lower in concentration than the effects observed in crystalline reaction zones and therefore had only a minimal effect on the experiments.

#### Supersaturation and nucleation rate calculations

Using the equations from Söhnel [40], Sangwal [35], and Pina and Putnis [23], supersaturation and nucleation rates were calculated for the above experiments. Briefly, the equation for supersaturation *S*(*x*) is:

$$S\left( x \right) = \sqrt {\frac{{a\left( {C^{2 + } } \right)^{1 - x} a\left( {B^{2 + } } \right)^{x} a\left( {A^{2 - } } \right)}}{{\left( {K_{CA} a_{CA} } \right)^{1 - x} \left( {K_{BA} a_{BA} } \right)^{x} }}}$$

where *B* represents Sr, Ba, or Pb of the crystal matrix; *C* represents Sr, Ba, or Pb in the added chloride solution, *A* = (SO_4_^2−^); K_CA_ and K_BA_ represent the appropriate solubility product constants at 60 °C; and *x* and (1 − *x*) represent the mole fractions of *B* and *C*, respectively. Solid solutions are assumed to be complete and ideal, simplifying the activity fractions *a*_*CA*_ and *a*_*BA*_ to 1. Concentrations, and subsequently activities, were estimated from the extrapolation/interpolation of data from various sources including Linke and Seidell [18], Krumgalz [16], initial experimental concentrations, and solubility products listed in Table 2. Experimental conditions prevented the possibility of measuring actual concentrations, mostly due to size constraints, so estimates were made based on solubilities of BaSO_4_, SrSO_4_, and PbSO_4_ in neutral chloride, acid chloride, neutral sulfate, and acid sulfate conditions at 60 °C. Although this does introduce some error, variation of the activities resulted in only minor changes in the trend curves produced—and even then, only in magnitude. The shape of the trend curves as well as the maximum X_BA_ values remained consistent.

**Table 2:** Solubility data for selected sulfates

Compound	*K*_sp_ (60 °C)	*V*_*mol*_ (m^3^)^d^	CIR^e^ (Å)	Sol (kg^−1^ H_2_O)
BaSO_4_^b^	2.216 × 10^−10^	8.67 × 10^−29^	1.75	3.62 mg (60 °C)
CaSO_4_∙2 H_2_O^b^	2.137 × 10^−5^	12.38 × 10^−29^	1.26	2559 mg (60 °C)
CaSO_4_∙0.5 H_2_O^b^	4.971 × 10^−5^	8.80 × 10^−29^	1.26	4212 mg (60 °C)
CaSO_4_^b^	1.674 × 10^−5^	7.64 × 10^−29^	1.26	1670 mg (60 °C)
SrSO_4_^b^	1.775 × 10^−7^	7.68 × 10^−29^	1.58	100 mg (60 °C)
PbSO_4_^a^	2.53 × 10^−8^	7.94 × 10^−29^	1.63	63.4 mg (60 °C)
RaSO_4_^c^	1.78 × 10^−10^	9.24 × 10^−29^	1.84	~ 4 mg (60 °C)

*CIR* crystal ionic radius of the cation (XII coordination) except Ca (VIII coordination)

Data sources: ^a^Haynes [12]; ^b^Krumgalz [16]; ^c^Brown et al. [3]; ^d^https://www.mindat.org, and references within; ^e^Shannon [39]

The nucleation rate function *J*(*x*) is calculated by:

$$J\left( x \right) = \varGamma \left( x \right)\exp \left[ {\frac{{ - B\sigma^{3} \left( x \right)\varOmega^{2} \left( x \right)}}{{k^{3} T^{3} \left( {\ln S\left( x \right)} \right)^{2} }}} \right]$$

where *Γ*(*x*) is the preexponential factor, estimated from molecular volume (Table 2); *Ω*(*x*) is molecular volume (Table 2); *σ*(*x*) is the interfacial free energy, estimated from K_sp_ values (Table 2); *B* represents a geometric factor dependent on nucleus shape; *k* is Boltzmann’s constant (1.38 × 10^−23^ J/K); *T* is temperature in Kelvin; and *S*(*x*) is the supersaturation factor from the equation above. A full description of these equations and their derivations can be found in Pina and Putnis [23] and references within. The reasoning behind these calculations is that thermodynamics alone do not always reproduce the observed results. Nucleation rates may significantly outweigh supersaturation ratios, and crystallization products may form contrary to solubility products [29].

#### Reactions in the presence of different sulfates

Two to five crystals of each compound (~ 200 μg) were placed together in 4.5 mL Exetainer glass screw-top vials. More or fewer crystals were added depending on size to roughly balance representation, but the samples were not weighed. Solutions (50 μL) were added to each vial. Table 3 lists the contents of the vials from experiments 1-4. Vials were capped and placed in a 60 °C oven for 30 h. After the allotted time, remaining crystals were rinsed while hot and sample preparation was completed in the same manner as above. Note that for this experiment all three cations are in direct competition in the same vials, and that the only source of Sr^2+^, Ba^2+^, and Pb^2+^ are from material dissolved from the crystals themselves.

**Table 3:** Experimental conditions for reaction experiments in the presence of different sulfates

Experiment/vial	Crystals	Solution	Approx. pH
1	Sr, Ba, Pb sulfates	0.1 M K_2_SO_4_	7
2	Sr, Ba, Pb sulfates	0.08 M H_2_SO_4_	1
3	Sr, Ba, Pb sulfates	0.1 M NaCl	7
4	Sr, Ba, Pb sulfates	0.12 M HCl	1

#### Crystal analysis using NanoSIMS

Samples were analyzed on the Cameca NanoSIMs 50L at the Centre for Microscopy, Characterisation, and Analysis (CMCA), located at the University of Western Australia, Perth, using previously established settings [33]. To best explore surface addition, replacement, or diffusive activity, sites near the edges of grains were mapped. A Hyperion (H200) RF plasma oxygen ion source was used for all analyses. The instrument was operated in multicollection mode, with five of the seven available detectors tuned to ^28^Si, ^40^Ca, ^88^Sr, ^138^Ba, and ^206^Pb. The additional two detectors were tuned to rare-earth element isotopes used in a separate experiment (and will thus not be referenced here). Maps of ^28^Si and ^40^Ca were included as quality control and to confirm that the silicon and calcium contribution to crystal growth was minimal. Similar instrument settings were used for all mapping (50 × 50 μm raster area, 50 pA ion current, D1 = 2, ES = 2, AS = 0, 512 × 512 px, 3 planes, 5 ms/px, effective beam diameter ≈ 400 nm).

Images were processed using ImageJ [36, 37] and the OpenMIMS plugin [25]. The color convention of Sr (in red), Ba (green), and Pb (blue) has been adopted for all images.

## Results and discussion

### Crystal characteristics

**Fig. 1:**
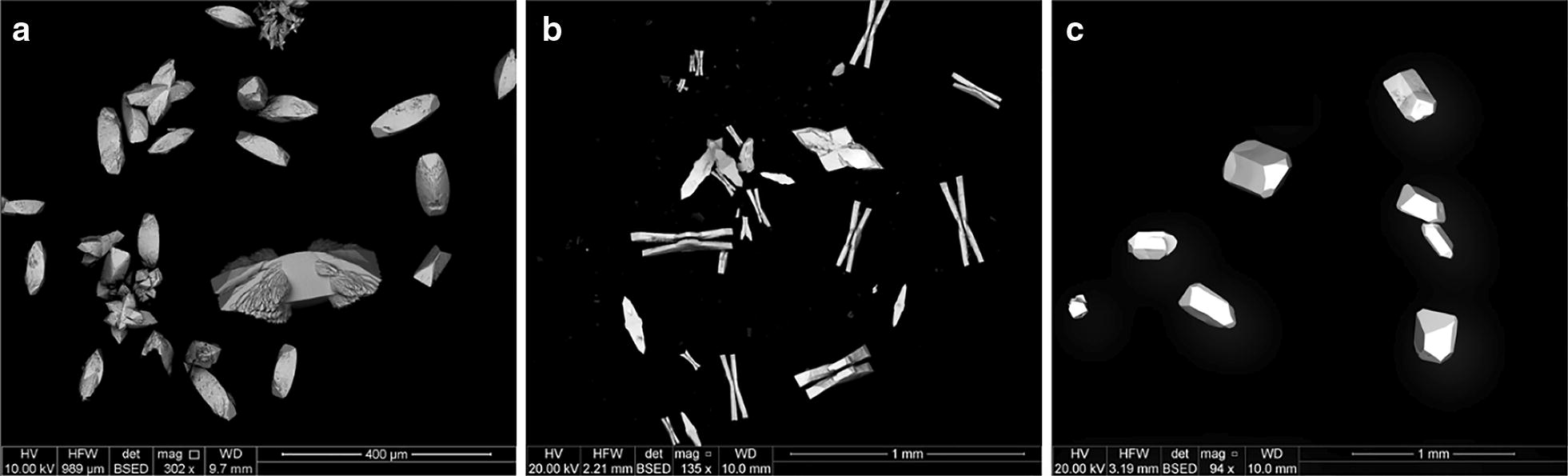
BSE images of laboratory-grown SrSO_4_ (**a**), BaSO_4_ (**b**), and PbSO_4_ (**c**)

All three sulfates crystallize in the orthorhombic crystal system, dipyrimidal (2/*m* 2/*m* 2/*m*) crystal class. SrSO_4_ formed slightly rounded orthorhombic prisms with dipyrimidal (chisel) terminations. Many crystals exhibited additional symmetric lateral growths (ears) near the prism/dipyramid interface. BaSO_4_ formed as double orthorhombic blades, centrally attached in bow-tie fashion. PbSO_4_ grew in well-formed euhedral prisms with varying dipyrimidal terminations. Figure 1 shows examples of the crystals produced from gel growth. Spectra of all three compounds were clean and sharp, with minimal traces of silicon found in the center of some of the grains but very little near the edges. This is not uncommon in crystals grown in a silica gel matrix, but nanoSIMS imaging revealed that this was not a factor in the experiment.

Although these synthetic crystals do not adhere to the strict definition of a mineral [8], the terms celestite, baryte, and anglesite are used interchangeably with SrSO_4_, BaSO_4_, and PbSO_4_, respectively, in the following sections. These crystals are designed to be simple but accurate proxies for the natural minerals in question, and evidence suggests that behaviors of the natural and artificial—with respect to these experiments—are aligned and would therefore apply equally to both.

### Leaching/recrystallization in simplified media

**Fig. 2:**
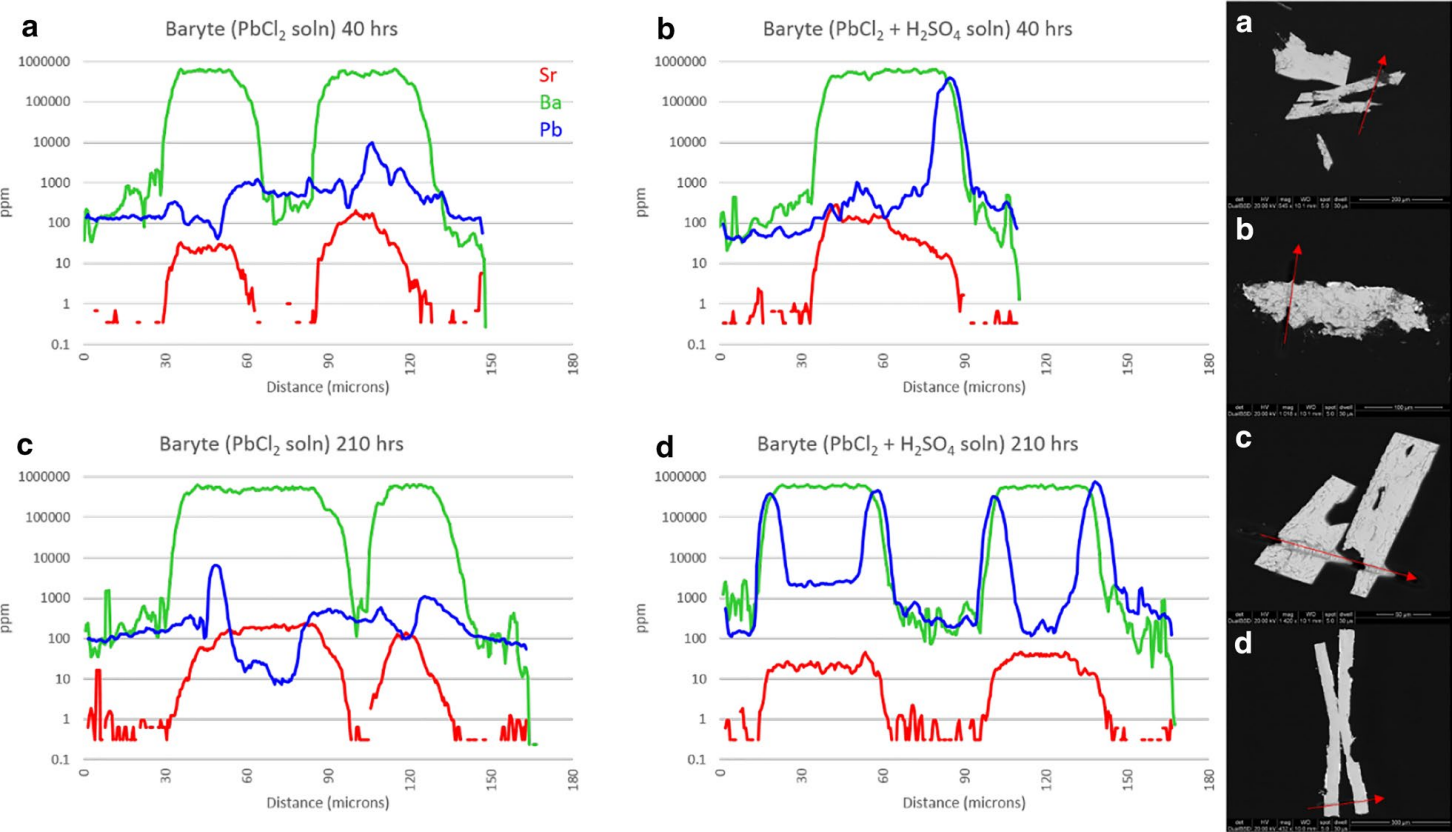
Results from experiment 5(a–d), baryte exposed to Pb^2+^ solution. LA-ICP-MS transects (**a**–**d**) correlate with BSE images (**a**–**d**). Red arrows show location and direction of laser ablation route

Figure 2 shows transects from barytes exposed to PbCl_2_ solution (experiments 5a–d), The BSE image of experiment 5d shows the typical bowtie morphology of the baryte crystals. The crystal in experiment 5b has tipped over and the bottom surface has been broken off. Thin, bright overgrowths of Pb-rich sulfate can be clearly seen in experiment 5b and d, with no visible surface effects in experiment 5a and c. The Sr background in the crystals is evident (everywhere < 200 ppm), with lower concentration areas the result of partial leaching.

Although reaction time seems to have had little effect on the experiment, the presence of sulfate has had a profound effect. Overgrowth of PbSO_4_ in experiment 5b and 5d occurs almost universally, though non-uniformly, up to 6 μm-thick in some areas. In transects, overgrowth is represented where the minor trace (Pb—blue) crosses over the major trace (Ba—green). All four surfaces of 5d show overgrowth in both BSE and LA-ICP-MS. Only one surface of experiment 5b shows the same, as the bottom surface was clearly broken during mounting. The roughly parabolic Pb traces appear wider than the overgrowth layers visible in the BSE image due to the relatively large beam width, with the increased Pb intensity beginning with the leading edge of the beam and ending with the trailing edge. The resulting width displayed by ICP-MS is the overgrowth layer plus the beam width plus any diffusive zone of the crystal face. Since the edge zones appear to be roughly symmetric (surface vs. interior), it is likely that diffusion of Pb into the BaSO_4_ structure was minimal. Crystals from experiment 5a and c show no evidence of Pb in BSE images, with relatively clean, sharp crystal surfaces. Transects of the same crystals indicate little to no uptake of Pb either as overgrowth on, or diffusion into, the crystal surfaces. The few enriched regions which are present may represent either limited uptake or surface contamination due to polishing, but the Pb concentrations there are nearly two orders of magnitude lower than those in the sulfate-available experiments.

**Fig. 3:**
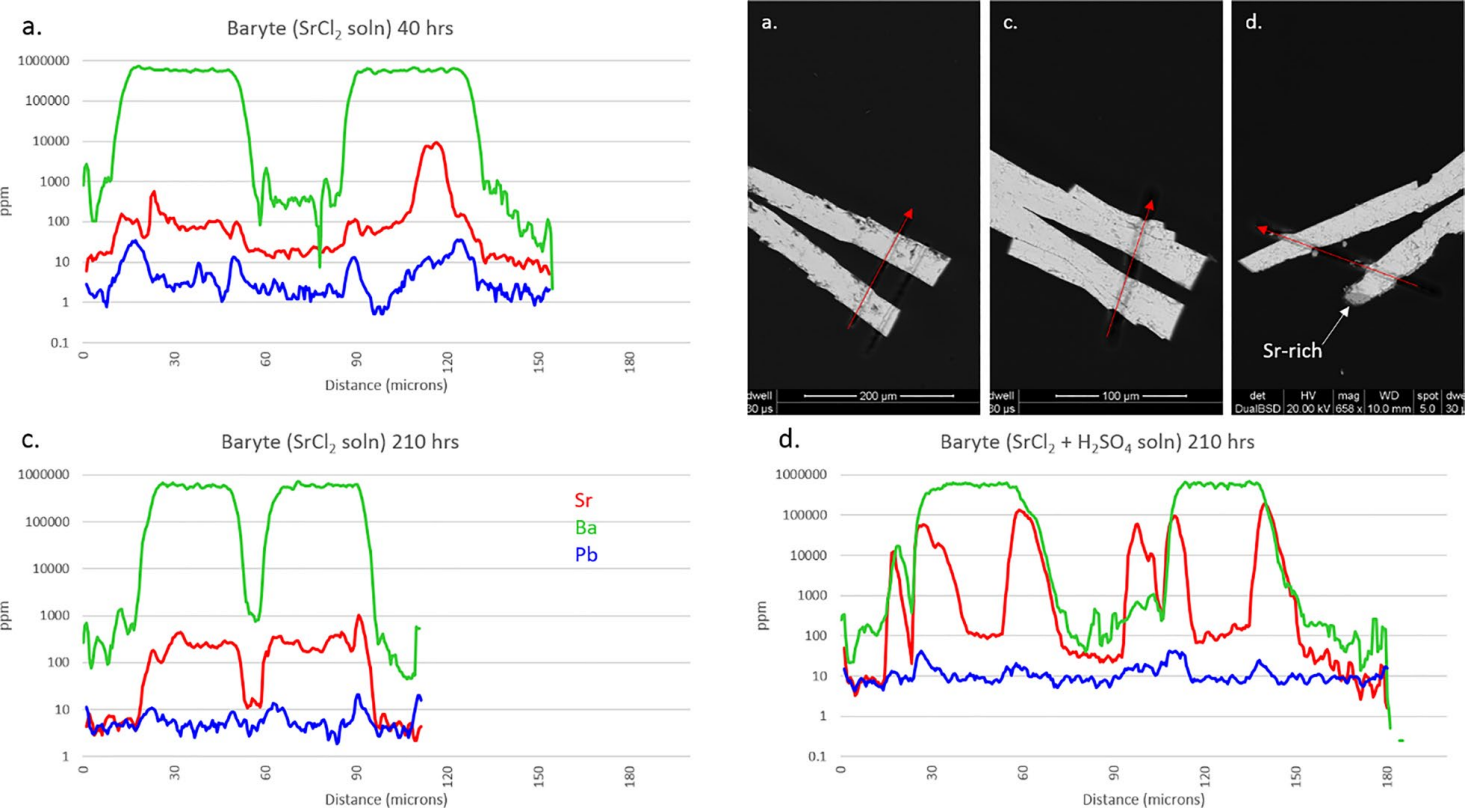
Results from experiment 6(a–d), baryte exposed to Sr^2+^ solution. LA-ICP-MS transects (**a**–**d**) correlate to BSE images (**a**–**d**). Red arrows show location and direction of laser ablation route. The baryte from vial 6b was lost

Barytes exposed to SrCl_2_ solution (experiments 6a, c, and d) are shown in Fig. 3. Unfortunately, the crystal from experiment 6b was lost. The Pb background in all crystals was consistently low, around 10 ppm, and had little impact on the experiment. The second laser transect visible in 6a was a test of instrument conditions. Although 6b is missing, it appears that the overall result of experiment 6(a-d) is similar to experiment 5(a–d), with reaction time having little effect but sulfate activity having a pronounced effect on the uptake of Sr. A dark overgrowth layer in 6d is clearly visible in the BSE image (to 15 μm-thick), although the ICP-MS data confirms that this layer is not pure SrSO_4_ but is predominantly BaSO_4_ with up to 20% Sr on a metals basis (m.b.). Consistent sloping of both Ba and Sr traces on the right side of both leaves indicates that the entire crystal is mounted in the epoxy at an angle, sloping upwards towards the “northwest” into the frame. No visible or measured Sr-rich edge zones are evident in either 6a or 6c, although one spot on the interior of 6a approaches 2% Sr.

**Fig. 4:**
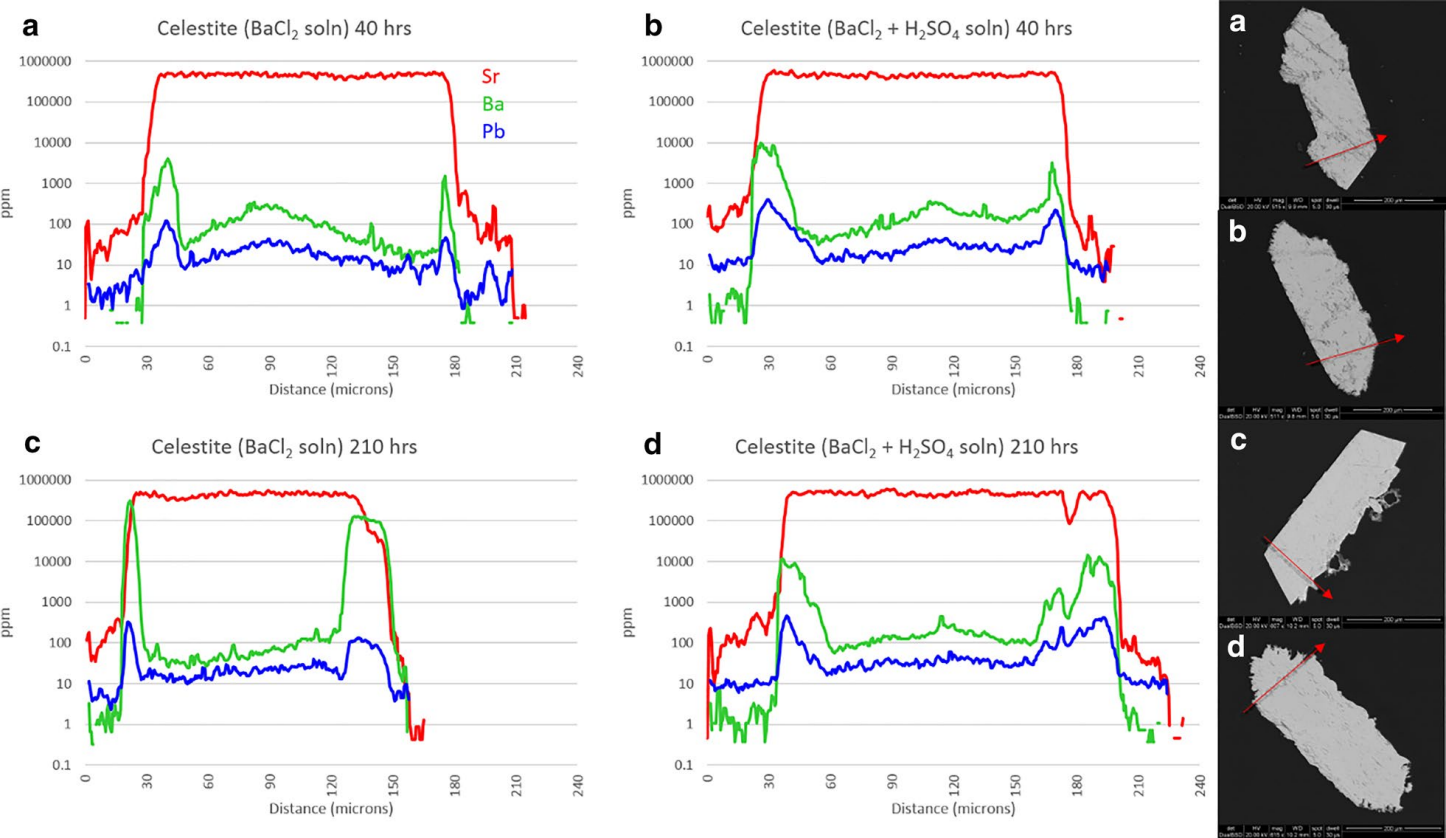
Results from experiment 7(a–d), celestite exposed to Ba^2+^ solution. LA-ICP-MS transects (**a**–**d**) correlate with BSE images (**a**–**d**). Red arrows show location and direction of laser ablation route

Figure 4 shows the results of experiment 7a–d (celestite exposed to BaCl_2_ solution). Pb in the crystal interiors generally ranges from 10 to 50 ppm, although it is suggested from edge zone concentrations (to 400 ppm Pb) that the BaCl_2_ solution probably contained some Pb. Uptake of Pb into grain edges mimics uptake of Ba but remains 1–3 orders of magnitude lower in concentration. All celestite crystals show visible porosity, the result of growth in a silica gel matrix. Slight enrichments in both Ba and Pb in grain centers are likely a result of this. Unlike the baryte experiments, it appears that reaction time does have some effect on the uptake of Ba by celestite, although this may only apply to low-sulfate activity conditions. Incorporation of Ba remains low at 40 h (< 1%, m.b.) but increases to > 70% in thin edge zones at 210 h. This may actually approach 100%, but ICP-MS resolution is limited by spot size. Crystals 7a and 7c maintain sharp features, and a bright Ba-rich replacement zone can be seen on BSE images for 7c although this rarely exceeds 1 μm in thickness. This is confirmed by ICP-MS as the Ba trace overlaps the Sr trace on both grain edges, with the right edge appearing more pronounced. This is likely from crystal mounting angle, suggested by the trailing edge of the laser transect on the right side of the crystal. Crystals 7b and 7d show signs of surface dissolution, amplified at terminations. Unlike the acid sulfate experiment from Phase 1, no overgrowth layers are visible. Independent of time, Ba uptake in high-sulfate activity conditions appears to stabilize around 2–2.5% (m.b.). Ba concentration appears to positively correlate with porosity in 7(b, d) but not 7(a, c), suggesting that freshly precipitated BaSO_4_ may be trapped in crystal pores.

**Fig. 5:**
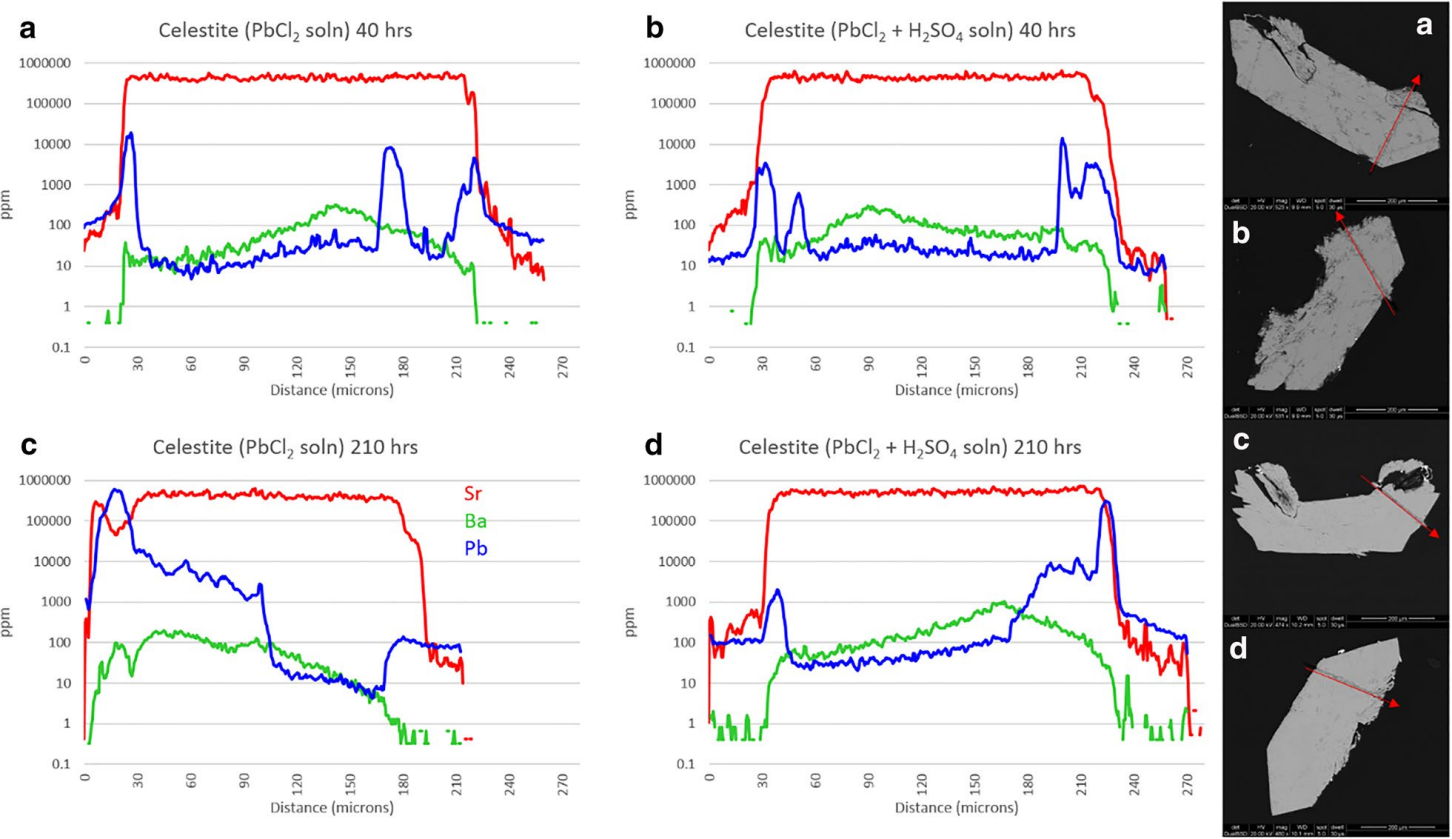
Results from experiment 8(a–d), celestite exposed to Pb^2+^ solution. LA-ICP-MS transects (**a**–**d**) correlate with BSE images (**a**–**d**). Red arrows show location and direction of laser ablation route

Transects of celestite exposed to PbCl_2_ solution (experiments 8a–d) are presented in Fig. 5. The impact of extraneous Ba is minimal, with slightly higher concentrations in crystal centers which is likely the result of porosity. All crystal faces are primarily sharp with little evidence of surface dissolution, although 8b has both clean and rough surfaces. Pb-rich sites are evident in all four BSE images, primarily as nucleated spots as opposed to uniform layers (although both exist). These spots may be freshly precipitated PbSO_4_ adhering to crystal surfaces or Pb-rich sulfates which grew during the experiment—it is very difficult to distinguish. Pb concentrations appear to be higher in longer experiments, with an increase from 3.5% Pb at 40 h to > 90% Pb at 210 h. Realistically, however, this may be the result of the laser transect crossing—or not crossing—a precipitated PbSO_4_ surface particle, so any broad statements about the effect of reaction time would be unsupported. In comparison, porosity clearly has a pronounced influence on Pb uptake, as seen from 8c. The left half of the crystal is enriched in Pb, which correlates perfectly with the high porosity region as seen in the BSE image. This is likely the result of enhanced incorporation of Pb due to increased surface area (and not the result of ineffective rinsing of residual PbCl_2_ solution from pores) as the ^35^Cl concentration remains minimal across both regions. With the exception of Pb-rich spots, it appears that maximum uptake of Pb remains around 2% regardless of reaction time or sulfate activity.

**Fig. 6:**
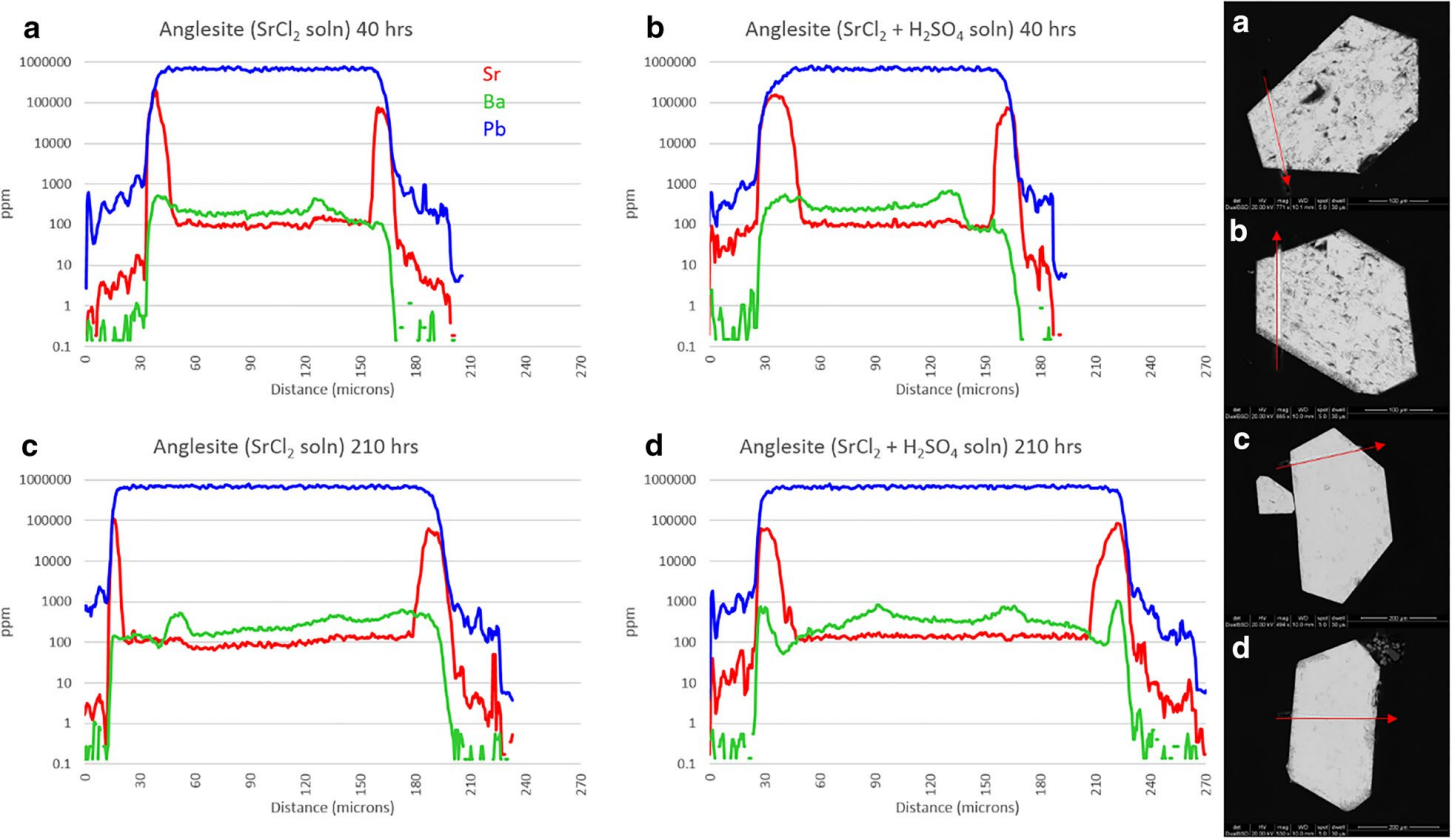
Results from experiment 9(a–d), anglesite exposed to Sr^2+^ solution. LA-ICP-MS transects (**a**–**d**) correlate with BSE images (**a**–**d**). Red arrows show location and direction of laser ablation route

Anglesite reactions with SrCl_2_ solution (experiments 9a-d) are presented in Fig. 6. The crystals remained euhedral, although some porosity is evident in 9a and 9b. This is likely due to growth rate, as the crystals which grew quickly (3–4 days) tended to have higher porosity than those which took 2 weeks or longer to form in the silica gel. Consistent concentrations of Sr in the center of all 4 grains is clear evidence that the extent of porosity had no effect on uptake here, in contradiction to the celestite experiments. Dark, Sr -rich rims are visible to some extent on all four crystals: on 9a and 9c as a thin, irregular layer (< 1 μm) on all surfaces; on 9b as thick (10 μm) replacement zones on the lower left and upper right surfaces $\left\{ {{\mathbf{101}},\;{{\bar{\bf 1}0\bar{\bf 1}}}} \right\}$ with an overlying 2 μm layer of very high-Sr sulfate covering every surface except {${\mathbf{101}}$}; and on 9d as very patchy Sr-rich replacement zones to 25 μm thick in some areas, but completely absent in others. As for the preferential reaction zones of sample 9b, anisotropic growth based on crystallographic orientation has been noted in baryte [24], and likely extends to the entire class of similar sulfates.

Neutral uptake of Sr into anglesite (9a, 9c) is sharp and thin, with the outer 1 μm layer surpassing Sr:Pb of 1.2. The Sr-rich zones of 9b also reach 1.2, but the ubiquitous thin overgrowth layer approaches 2.8. On 9d, however, the diffuse Sr-rich regions only contain about 30% Sr (m.b.). Interestingly, Sr concentrations in grain edges seem to stabilize around 100,000 ppm independent of sulfate activity, reaction time, or reaction zone width.

**Fig. 7:**
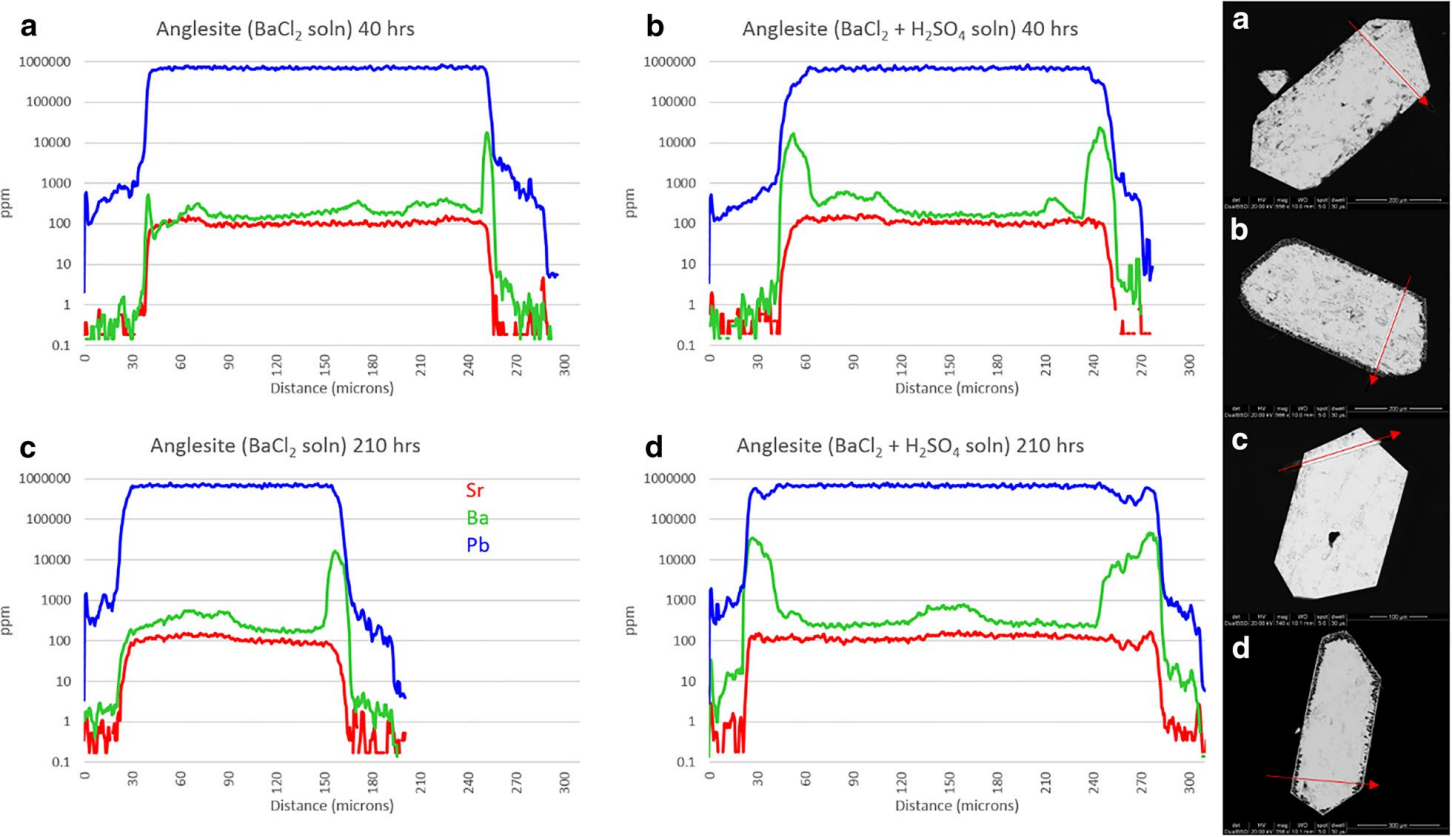
Results from experiment 10(a–d), anglesite exposed to Ba^2+^ solution. LA-ICP-MS transects (**a**–**d**) correlate with BSE images (**a**–**d**). Red arrows show location and direction of laser ablation route

Figure 7 shows anglesite reactions in Ba solutions (experiments 10a-d). Background Sr concentrations remain a consistent 100 ppm and do not affect the results. Crystals from experiments 10a and 10c are still euhedral and sharp, with no uptake or overgrowth of Ba visible in BSE images. Acid-leached crystals, however, show signs of significant surface reactions with rounded edges and dissolution/recrystallization textures extending up to 40 μm deep. LA-ICP-MS transects show Ba uptake in these reaction zones, but only to about 3 wt%. Time seems to have played a minor role, with Ba concentrations only slightly increased in 210-h experiments.

### Interpretation and discussion

For baryte in experiment 5a–d, the *S*(*x*) and *J*(*x*) calculations are in agreement with experimental observations. With access to only PbCl_2_, supersaturation remains low and nucleation rate is so low as to be effectively zero. Accordingly, virtually no Pb is diffused into, or crystallized on the surface of, baryte (Fig. 2a, c). In PbCl_2_ + H_2_SO_4_, however, the supersaturation function is significantly higher with a maximum of 100 at X_BaSO4_ = 0.66. Although this may imply that BaSO_4_ crystallization would be slightly favored, the nucleation rate maximum of ~ 1.2 × 10^14^ nuclei/cm^3^s is actually found at X_PbSO4_ = 0.9 indicating that PbSO_4_ nucleation is heavily favored. Figure 2b, d shows precisely this with thick, nearly pure PbSO_4_ layers grown (albeit unevenly) on baryte surfaces.

Barytes from experiment 6a-d also correlate to the models, with similarly low *S*(*x*) and *J*(*x*) values for sulfate-free environments and significantly higher *S*(*x*) and *J*(*x*) values in sulfuric acid solutions. Correspondingly, thick zones of Sr-rich sulfate can be seen on baryte surfaces in Fig. 3d.

Celestite models behaved somewhat differently from baryte, although similarities can be seen between the high-sulfate (Ba, Sr)SO_4_ trends and (Sr, Ba)SO_4_ trends. With exposure to BaCl_2_, higher supersaturation functions occur for both solutions, both heavily favoring BaSO_4_. Nucleation rates are split, with sulfate-free solutions favoring BaSO_4_ precipitation and sulfuric acid solutions preferring SrSO_4_ precipitation. Experimentally, Ba remains low in celestite with < 1% (m.b.) being common although thin BaSO_4_-rich overgrowth zones are seen in Fig. 4c. Nucleation rates would suggest that in sulfate-rich environments SrSO_4_ is more likely to precipitate than BaSO_4_, although some Ba uptake is inevitable providing there is some Ba^2+^ available. Opposite inclinations exist in sulfate-poor environments. The former is counter-intuitive considering the higher solubility of celestite, but is confirmed experimentally by the presence of an irregular BaSO_4_ layer on the celestite surface in Fig. 4c—and the complete lack of the same in Fig. 4b, d, with the minimal Ba signal in the latter being due to diffusion, rather than an overgrowth of BaSO_4_.

Lead and Sr sulfate have similar solubility products; therefore, it is expected that nucleation rates will be similar. Celestite exposed to PbCl_2_ solution exhibits favorability towards PbSO_4_ in both the *S*(*x*) and *J*(*x*) functions, but the addition of H_2_SO_4_ not only greatly increases the nucleation rate but also shifts the *J*(*x*) maximum from pure PbSO_4_ to a more predictable X_PbSO4_ = X_SrSO4_. Figure 5c clearly shows a few microns of PbSO_4_ overgrowth on the celestite surface, although the same cannot be seen in Fig. 5a. Pb uptake by celestite in sulfate solutions seems to remain low, although there are a few PbSO_4_ particles on the crystal surface in Fig. 5d. These are likely to be precipitates from the initial solution mixing which became lodged in the porous surface of the celestite, as no layering or adhesion to the surface is apparent.

Not surprisingly, *S*(*x*) and *J*(*x*) trends for the (Pb,Sr)SO_4_ system is similar to the (Sr,Pb)SO_4_ system. With less Pb^2+^ available, the *S*(*x*) and *J*(*x*) values for the Pb endmembers have been reduced to nearly zero in the sulfate-free solution. *S*(*x*)_*max*_ and *J*(*x*)_*max*_ in sulfuric acid solution remain the same, at X_PbSO4_ ≈ 0.8 and 0.5, respectively, although the nucleation rate is somewhat reduced. From the LA-ICP-MS data, it can be seen that Sr^2+^ uptake by anglesite occurs in both systems, but different mechanisms may be responsible depending on SO_4_^2−^ availability. [Sr] seems stable at ~ 10 wt% in all four crystals in Fig. 6a–d, but the sulfate-free samples show little to no surface alteration in the BSE images, whereas the sulfuric acid samples are clearly altered in zones up to 30 μm thick. The simplest explanation is that—despite very low nucleation rates—very thin zones of high-Sr sulfate is forming on anglesite in sulfate-free solutions (overgrowth), whereas thick, porous zones of mixed Pb,Sr sulfates are replacing anglesite in sulfate-rich solutions (CDR).

Keeping with the trend, *S*(*x*) and *J*(*x*) curves for (Ba,Pb)SO_4_ are similar to those for (Pb,Ba)SO_4_. Although supersaturation seems to favor BaSO_4_ precipitation, nucleation rates suggest nearly pure PbSO_4_ will occur instead. This effect presents as thick layers of PbSO_4_ on baryte but exhibits as a lack of any precipitation (or a reprecipitation of Ba-doped PbSO_4_) on anglesite. Little to no Ba is present in the crystals shown in Fig. 7a, c, achieving only 1 wt% in very thin zones not visible in BSE images. With H_2_SO_4_, there is clearly a CDR reaction occurring, but the precipitation rate seems to be lagging significantly behind the dissolution rate. Porosity is very high, with most of the sparse replacement sulfate being Pb-dominant.

These are obviously simplified models and although nucleation rate predictions seem to parallel experimental results there are other factors to consider. Chloride complexation amplifies Sr-, Ba-, and PbSO_4_ solubilities by many orders of magnitude, and are only passively included in the kinetics calculations in the form of sulfate solubility estimates in Cl^−^ solutions. It is also well known that both thermodynamic and kinetic properties tend to diverge between bulk systems and nanoscale structures such as fractures [41, 42], pores [28], and ultrathin fluid–solid reaction interfaces [1].

### Leaching/recrystallization in experiments involving multiple sulfates

#### Experimental results

**Fig. 8:**
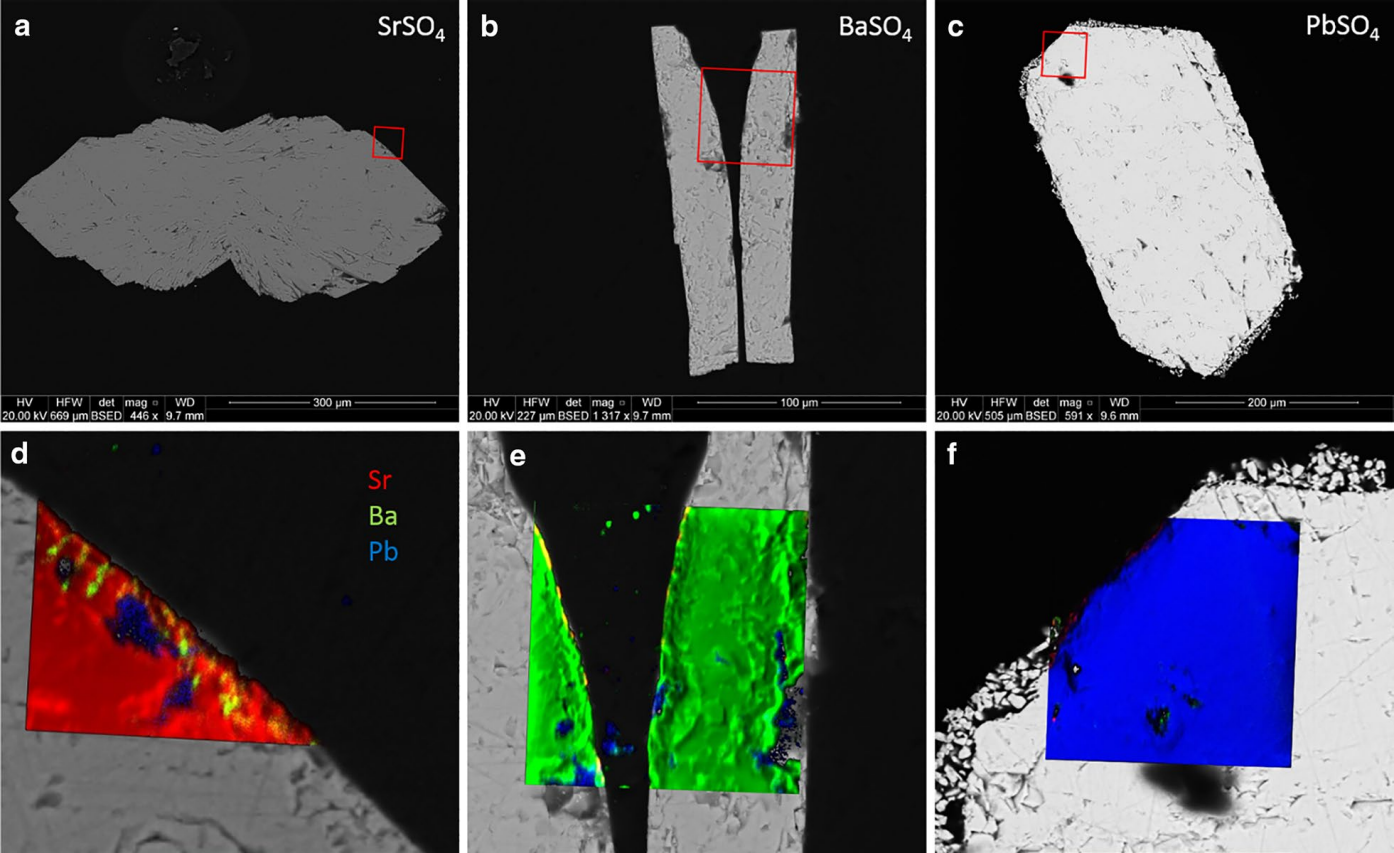
BSE (**a**–**c**) and nanoSIMS overlays (**d**–**f**) of crystals exposed to neutral sulfate solution (vial 1). Sr = red, Ba = green, Pb = blue

Most of the crystals from experiment 1 (neutral sulfate solution) were still clear and euhedral after the experiment. One PbSO_4_ crystal suffered damage during removal from the vial which resulted in a detrital coating on the undamaged PbSO_4_ crystal, visible in the BSE image. Figure 8 shows BSE images of the crystals (top) as well as color composites of the ^88^Sr, ^138^Ba, and ^206^Pb nanoSIMS distribution maps overlying zoomed-in sections of the BSE images (bottom). Red squares indicate the area of mapping. The crystals appear to be internally clean, although small amounts of Pb are seen filling near-edge gaps in both the SrSO_4_ and BaSO_4_ crystals. Small patches of Ba can be seen within a few microns of the edge of the SrSO_4_, and a trace amount of Sr can be seen on the inside edge of the left crystal of BaSO_4_ (appears as orange). Virtually no Sr or Ba was detected in or on the surface of the PbSO_4_.

**Fig. 9:**
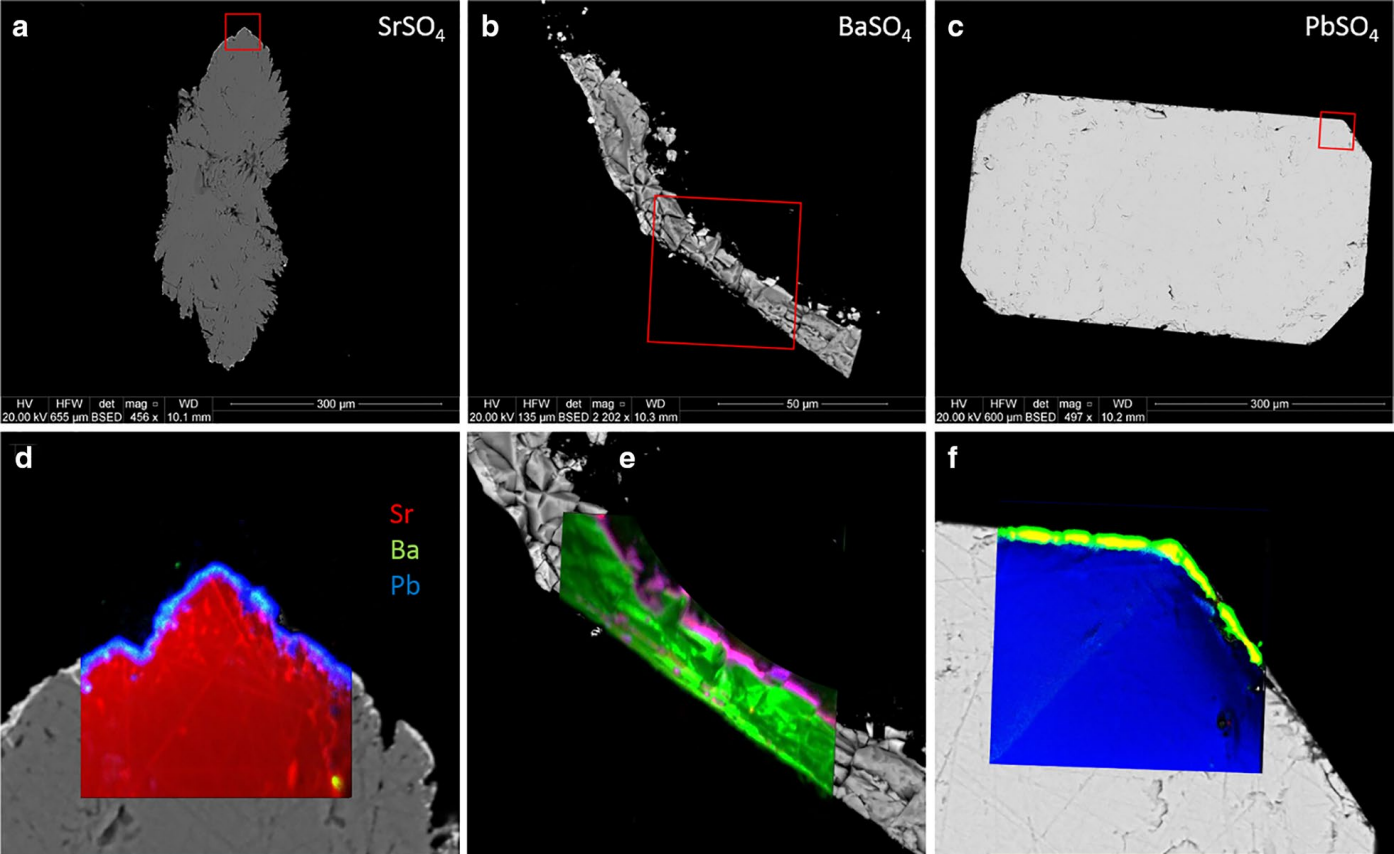
BSE (**a**–**c**) and nanoSIMS overlays (**d**–**f**) of crystals exposed to acid sulfate solution (vial 2). Sr = red, Ba = green, Pb = blue. The thin magenta edge in (**e**) indicates Sr–Pb overlap

Figure 9 shows the resulting crystals from experiment 2 (acidic sulfate solution). These crystals were also in very good condition visually, although the normally transparent PbSO_4_ had become translucent. The BaSO_4_ crystal shattered during polishing. BSE images show a thin veneer of overgrowth on all three crystals, consisting of the other two sulfates. NanoSIMS images confirm this, and clearly show a thin (< 2 μm) layer of recrystallization. A comparison of signal intensities of the rim on SrSO_4_ produces an estimated Sr:Ba:Pb ratio of 15:1:10; for the BaSO_4_ rim the ratio is 4:6:9; for the PbSO_4_ rim the ratio is 8:11:2.

**Fig. 10:**
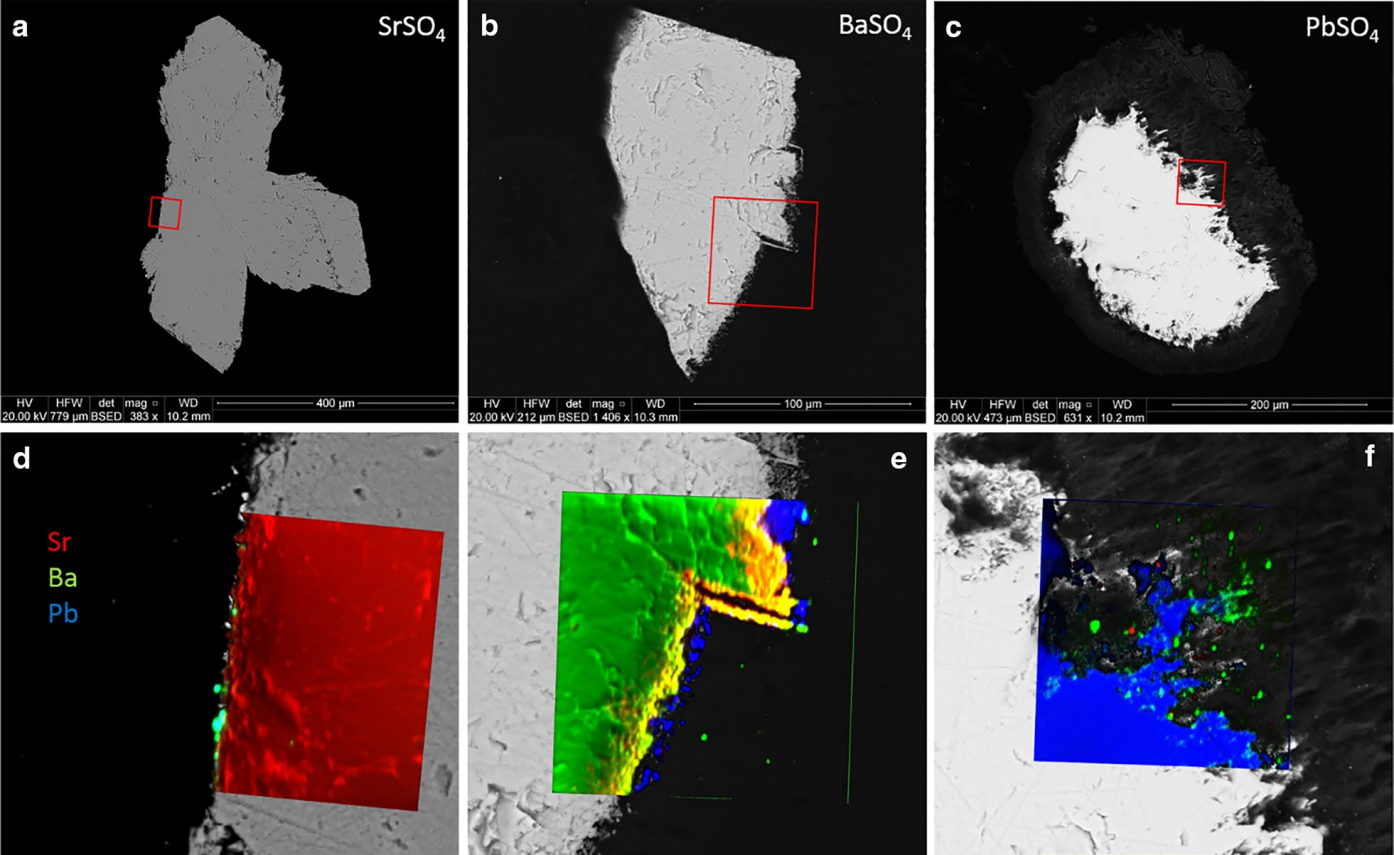
BSE (**a**–**c**) and nanoSIMS overlays (**d**–**f**) of crystals exposed to neutral chloride solution (vial 3). Sr = red, Ba = green, Pb = blue. The yellow-orange band in (**e**) denotes Sr–Ba overlap

The BaSO_4_ and PbSO_4_ crystals from experiment 3 (neutral chloride solution) were noticeably corroded and opaque, and thin layers of overgrowth could be seen flaking off even under low magnification. SrSO_4_ appears to have survived quite well, although surface pitting was visible under magnification. BSE images (Fig. 10) show some etching of BaSO_4_ and significant dissolution of PbSO_4_. NanoSIMS mapping revealed very little recrystallization on the SrSO_4_ and PbSO_4_ but showed what appeared to be Sr diffusion up to ~ 6 μm into the BaSO_4_ crystal with a thin, pure PbSO_4_ layer on the surface.

**Fig. 11:**
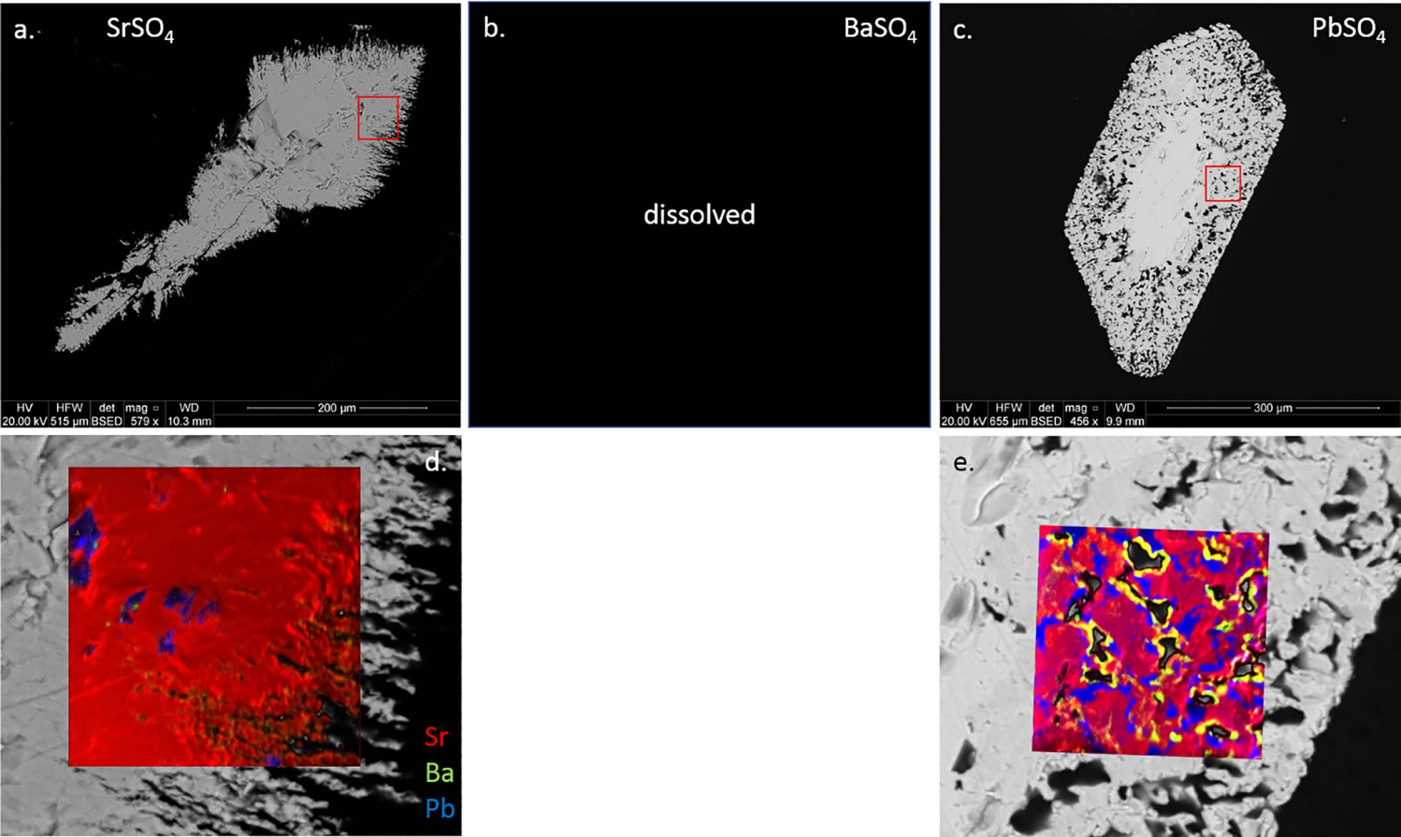
BSE (**a**–**c**) and nanoSIMS overlays (**d**–**e**) of crystals exposed to acid chloride solution (vial 4). Sr = red, Ba = green, Pb = blue. The yellow edging in (**e**) indicates Sr–Ba overlap

The surviving crystals from experiment 4 (acid chloride solution) were significantly corroded and opaque. No BaSO_4_ crystals were found, having presumably complexed with excess chloride and dissolved. NanoSIMS maps show little to no deposition on the surface of the corroded SrSO_4_, but the PbSO_4_ shows significant dissolution and replacement by both BaSO_4_ and SrSO_4_. Despite extensive Pb removal, the crystal clearly retained its original shape and the PbSO_4_ core remains intact. Figure 11 illustrates the extent of replacement, as the original PbSO_4_ (blue) is virtually gone with Sr (red) nearly completely replacing Pb, and imported Ba (green, but displays as yellow-orange where overlapping red color) highlighting the edges of exposed surfaces.

**Fig. 12:**
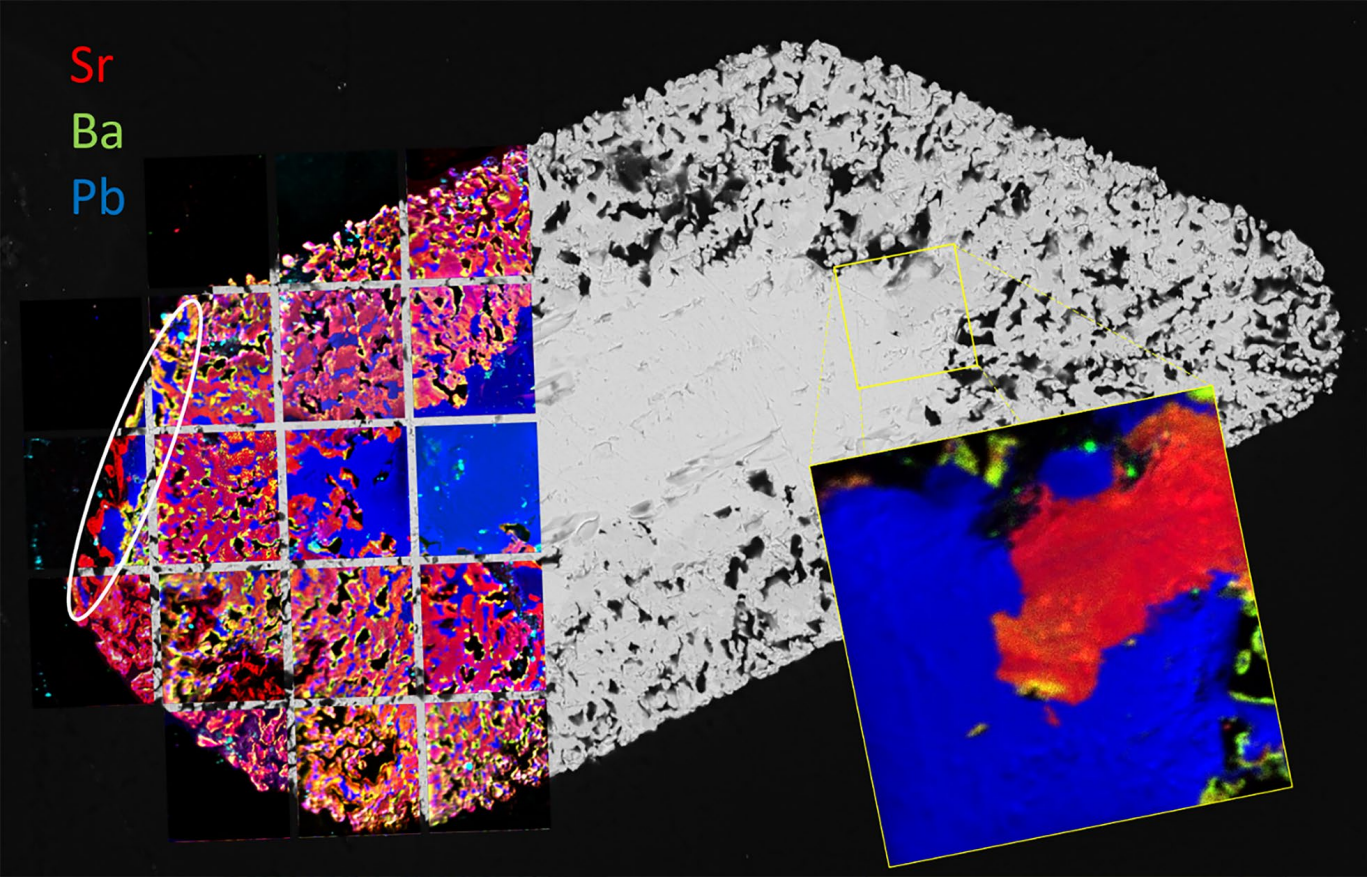
BSE image of PbSO_4_ grain B4, overlain with 19 tri-color composite nanoSIMS images. Sr = red, Ba = green, Pb = blue

**Fig. 13:**
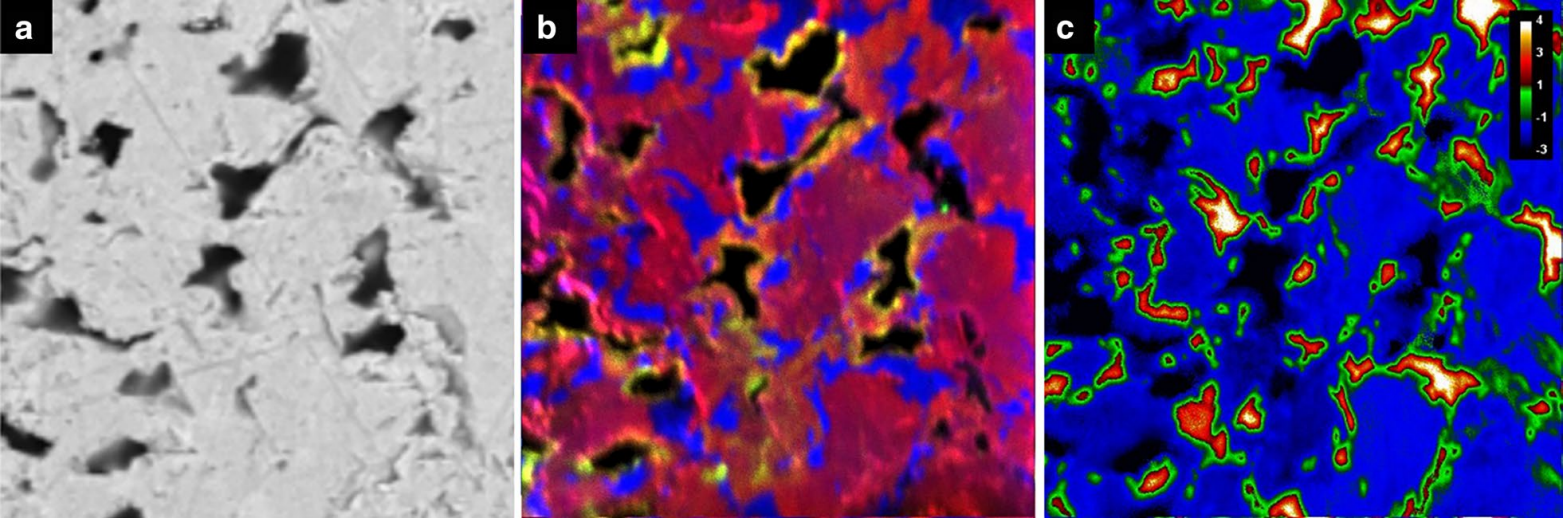
Detail of a 50 × 50 μm area from PbSO_4_ grain B4, shown as **a** BSE image, **b** color composite (Sr = red, Ba = green, Pb = blue), and **c** log Pb:Sr. The yellow edging in **b** represents Sr–Ba overlap

Figure 12 contains a montage of 18 nanoSIMS images of the same grain, B4. The extent of Sr replacement is evident, as is the remaining untouched core of pure PbSO_4_. A remnant strip of PbSO_4_ can also be seen near the left edge of the grain (circled). The inset image shows detail of the boundary between the original PbSO_4_ and invasive Sr. Despite appearances, regions of Sr replacement still contain lead, and semi-quantification shows this material to be close to 3:1 in Sr:Pb. The 3:1 ratio holds for replacement regions near the edge as well as in the interior of the crystal. Similar analyses of blue regions reveal nearly pure PbSO_4_, with Pb:Sr being > 100. Figure 13 shows a small area of grain B4, with BSE (a), color-composite map (b), and log-scale distribution map of Pb:Sr (c). Although the color composite seems to indicate great variation in composition, the Pb:Sr map shows that there are primarily three distinct classifications: high-Pb (red to white); high-Sr, low-Pb (blue); and epoxy (black). Boundaries between the relatively pure PbSO_4_ regions and the Sr:Pb ≈ 3 regions are quite sharp, and the thin green perimeters are likely to be a result of a relatively large beam width (~ 400 nm) compared to pixel size (~ 100 nm) and not true gradients. Barium contribution is minimal, with the brightest area on the image representing only about 25% Ba content (total cations).

**Fig. 14:**
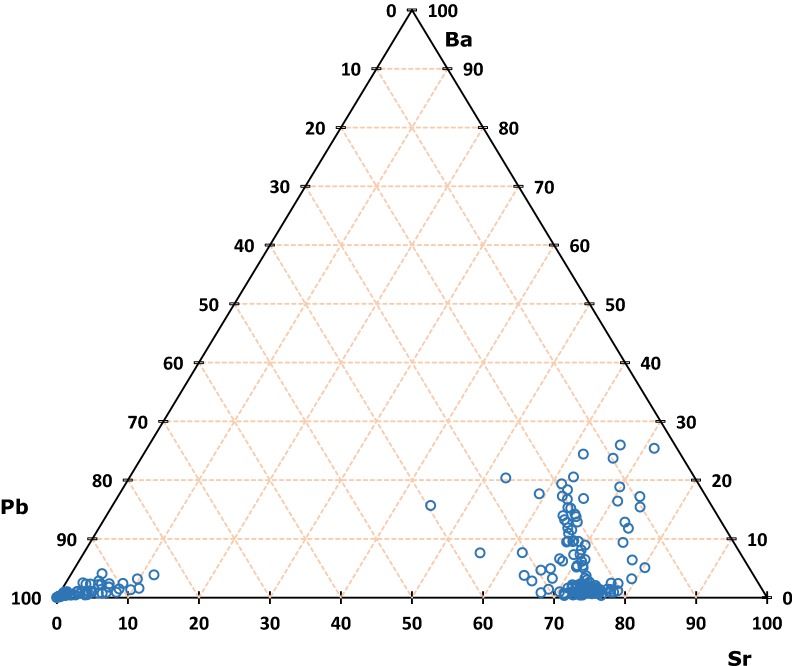
Ternary Ba-Pb-Sr diagram of 225 nanoSIMS spot analyses from grain B4. Ba:Sr:Pb in wt%, normalized to 100%

Figure 14 contains Sr:Ba:Pb ratios (in wt%, normalized to 100%) for 225 point analyses of nanoSIMS data from throughout the composite image in Fig. 12. Three classifications appear here, as well, with most of the points clustering around a rough composition of Sr_3_Pb(SO_4_)_4_. A second cluster represents samples of nearly pure lead sulfate, with some samples trailing into the “Sr-bearing” zone. Again, this is quite possibly due to beam-width overlap as the true boundaries appear to be distinct and sharp. The third classification of points represents areas containing > 5% Ba, found almost exclusively near exposed surfaces. These regions likely crystallized last and contain mostly SrSO_4_ but may contain up to 55% (Pb + Ba)SO_4_.

### Interpretation and discussion

The results shown in Fig. 8 (experiments containing neutral sulfate solution), are no surprise. Of the entire periodic table, only Ba, Ca, Pb, Ra, and Sr form insoluble sulfates in their most common oxidation state, with minor species including Hg^+^, Ag^+^, and Po^4+^ [11]. The lack of available H^+^ and of any complexing agent results in low activity and therefore virtually no mobility, with the compounds’ solubility in water being the effective limiting factors. For a 200 μg sample, the most soluble of these, SrSO_4_, should only lose a maximum of 3% to dissolution at 60 °C.

In Fig. 9, crystals from experiment 2 (0.08 M acid sulfate solution) show increased surface activity, with movement between all three compounds. A thin veneer of overgrowth < 2 μm thick can be seen in the BSE images, especially around the SrSO_4_. Slight pitting and etching can also be seen, although this does not penetrate the crystal surfaces more than a micron or two. NanoSIMS images confirm that a thin layer of (Sr,Ba,Pb)SO_4_ has deposited on the surface of all three crystals, although the Sr:Ba:Pb ratio differs between the three. From these ratios, it appears that Sr mostly either precipitates back on the SrSO_4_ as a mixed Sr-Pb sulfate, with little Ba, or it precipitates on the PbSO_4_ as a Sr-Ba mixed sulfate, with very little Pb. Lead mobility seems to be one-directional, with liberated Pb^2+^ preferring to recrystallize on the other two sulfates but not back on the PbSO_4_.

LA-ICP-MS results seem to differ here from the nanoSIMS maps, with thin overgrowths evident in experiments 1-4 but only on both barytes and one anglesite in the simple reaction experiments. Although both sets of experiments were under acid sulfate conditions, those in the simple reaction experiments also contained significant chloride from the added 0.1 M MCl_2_ solutions. In effect, experiment 2 contained only H_2_SO_4_ whereas the “b” and “d” sub-set of experiments 5 through 10 contained [H^+^] + [SO_4_^2−^] + [Cl^−^]. Chloride is well known as a strong complexing agent with Sr, Ba, and Pb which appears to have subdued the formation of overgrowth layers in some cases, especially on celestite.

Results for crystals of BaSO_4_ and PbSO_4_ in Fig. 10 from experiment 3 (0.1 M neutral chloride solution) were similarly unsurprising, as it is well known that alkali earths and lead form soluble chloride complexes. Visible etching of the SrSO_4_ was surprisingly low, however Lucchesi and Whitney [20] show that for < 1 M NaCl, solubility of SrSO_4_ decreases with temperature, with values of 185 mg/L at 0 °C and 170 mg/L at 25 °C. An estimate of 150 mg/L for 60 °C would result in < 4% solubility for a 200 μg sample. The BaSO_4_ shows noticeable etching and pitting on the surface. The small amount of Sr^2+^ liberated seems to have ended up entirely in the BaSO_4_, appearing as a diffuse boundary extending up to 6 μm in from the surface. Although a substantial amount of Ba^2+^ has been liberated from the sulfate, none of this is found in the SrSO_4_—although a few tiny blebs can be seen on the surface. With crystal ionic radii (CIR) of 1.58 Å for Sr^2+^ and 1.75 Å for Ba^2+^ [39], it makes sense that BaSO_4_ could incorporate Sr^2+^ while SrSO_4_ would not accommodate the larger Ba^2+^. Pb^2+^ is very similar in size to Sr^2+^, having a CIR of 1.63 Å (ibid.). There is a thin, patchy layer of pure PbSO_4_ on the surface of the BaSO_4_. It is logical that most of the Pb and Ba liberated by complexation with chloride remained in solution and was rinsed away when the crystals were washed.

Laser ablation results from the “a” and “c” sub-sets of experiments (also neutral chloride conditions) expand on these results. Thin Ba and Pb overgrowth layers are seen on celestite but only in the 210-h experiments, indicating sluggish kinetics. Baryte crystals showed little uptake of either Sr or Pb, but independent spots did reach 1 wt% in both cases. Anglesite showed an affinity towards Sr, incorporating over 100,000 ppm in thin edge zones. This was not apparent from the nanoSIMS results, the primary difference being that the only source of Sr^2+^ in that experiment was the SrSO_4_ crystal itself—which showed virtually no signs of dissolution. When Sr^2+^ is in great excess, however, anglesite readily accommodates thin Sr-rich overgrowth layers. Barium uptake in anglesite is negligible with Ba:Pb never exceeding 0.25, and then only in sub-micron patchy layers.

Experiment 4 (0.12 M acid chloride solution) gave the most impressive results. BaSO_4_ had completely complexed with chloride and was removed with the rinse. This was surprising considering the relatively moderate removal of BaSO_4_ in the neutral chloride solution. Little work has been done comparing neutral vs. acidic solutions of the same anion, but Lucchesi and Whitney [20] show for SrSO_4_ that pH has little effect on solubility in sulfate solutions but that HCl can dissolve 3–3.5 times as much SrSO_4_ as similar concentrations of NaCl. For baryte, the K_sp_ would have to increase by more than 3 orders of magnitude to account for complete removal. The crystal ionic radius of Ba^2+^ would prevent diffusive uptake into either PbSO_4_ or SrSO_4_, although some late-stage replacement (co-crystallization with Sr) of PbSO_4_ seems apparent from the nanoSIMS images.

Liberated Sr^2+^ appears to have predominantly replaced Pb^2+^ in the PbSO_4_ crystal. Thin BaSO_4_-rich rims are found on most surfaces (Fig. 13b), indicating either a local (fluid-crystal interface) or “global” increase in [Ba^2+^]/([Pb^2+^]+[Sr^2+^]). Local concentration gradients may exist due to a lack of convection, with interfacial fluid chemistry following different thermodynamic properties from bulk fluids [10, 15, 34]. Global concentration changes would imply precipitation of sufficient Pb and Sr from the overall solution to trigger favorable kinetics for BaSO_4_ (or at least Ba-rich sulfate) precipitation. Visible textures suggest coupled dissolution/reprecipitation (CDR), a mechanism of pseudomorphic replacement [26, 27, 30] with the possible end-result (if given sufficient time) being a celestite pseudomorph after anglesite. Future experiments will test this pathway further.

### Synthesis and comparison of results

Laser ablation results from the “b” and “d” sub-set of experiments 5-10 show remarkably similar patterns to those in experiment 4—at least for celestite and anglesite. Incorporation of Ba and Pb into celestite is minimal, topping out at 1.2 wt% and 1.4 wt%, respectively. Etching of surface material is visible. Baryte showed more uptake, with thick overgrowth layers of nearly pure SrSO_4_ (Sr:Ba approaching 7) and PbSO_4_ (Pb:Ba surpassing 150). Baryte dissolution in experiment 4 is likely the result of that vial containing HCl with no added (SO_4_^2−^), whereas experiments 5(b,d) and 6(b,d) contained H^+^, Cl^−^, and excess (SO_4_^2−^). Excess sulfate will affect activity factors as well as inhibit dissolution, even in the presence of chloride. Anglesite once again showed the most impressive results, with thick, visible zones of dissolution and recrystallization in both Sr^2+^ and Ba^2+^ solutions. Although the Sr:Pb did reach 2.7 on one edge, in agreement with the nanoSIMS results, most areas showed lower—but still significant—concentrations of Sr. This is likely due to excess sulfate in in the simple reaction experiments, while experiment 4 contained excess acid chloride only. Additionally, dissolution of all three was more significant in the multi-sulfate experiments for the same reason.

These results indicate there are three distinct mechanisms at work, depending on conditions: overgrowth, diffusion, and CDR. Overgrowth refers to the addition of crystalline sulfate on the surface of an existing crystal which has a different composition than the host. The pure PbSO_4_ layer on the surface of baryte in Fig. 2d is an excellent example. In this case, the BaSO_4_ structure acts as a template for PbSO_4_ crystallization, but there appears to be no dissolution of baryte or significant incorporation of Ba^2+^ in the fresh PbSO_4_ layer. The morphology of the parent crystal is not maintained, with the PbSO_4_ clearly a surficial addition. There is also no porosity visible in the PbSO_4_.

**Fig. 15:**
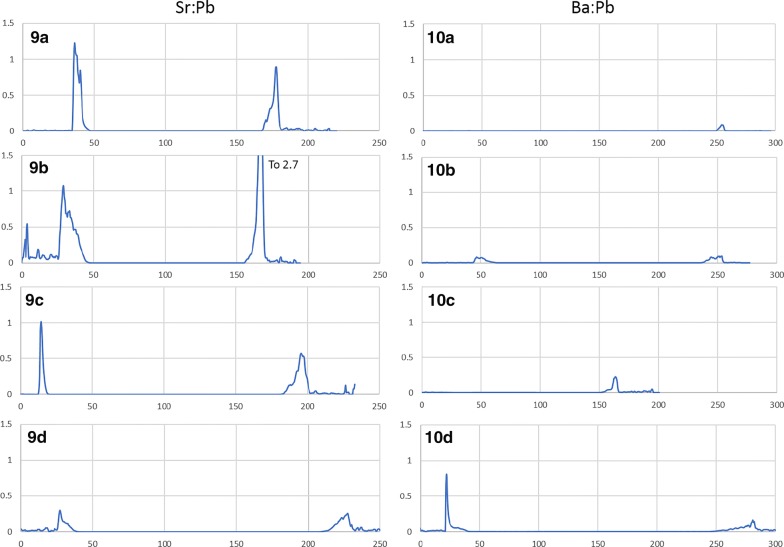
Ratios of [Sr]:[Pb] from experiment 9(a–d) and of [Ba]:[Pb] from experiment 10(a–d). X-axes (in μm) are identical to the corresponding LA-ICP-MS transects above (Figs. 6 and 7)

**Fig. 16:**
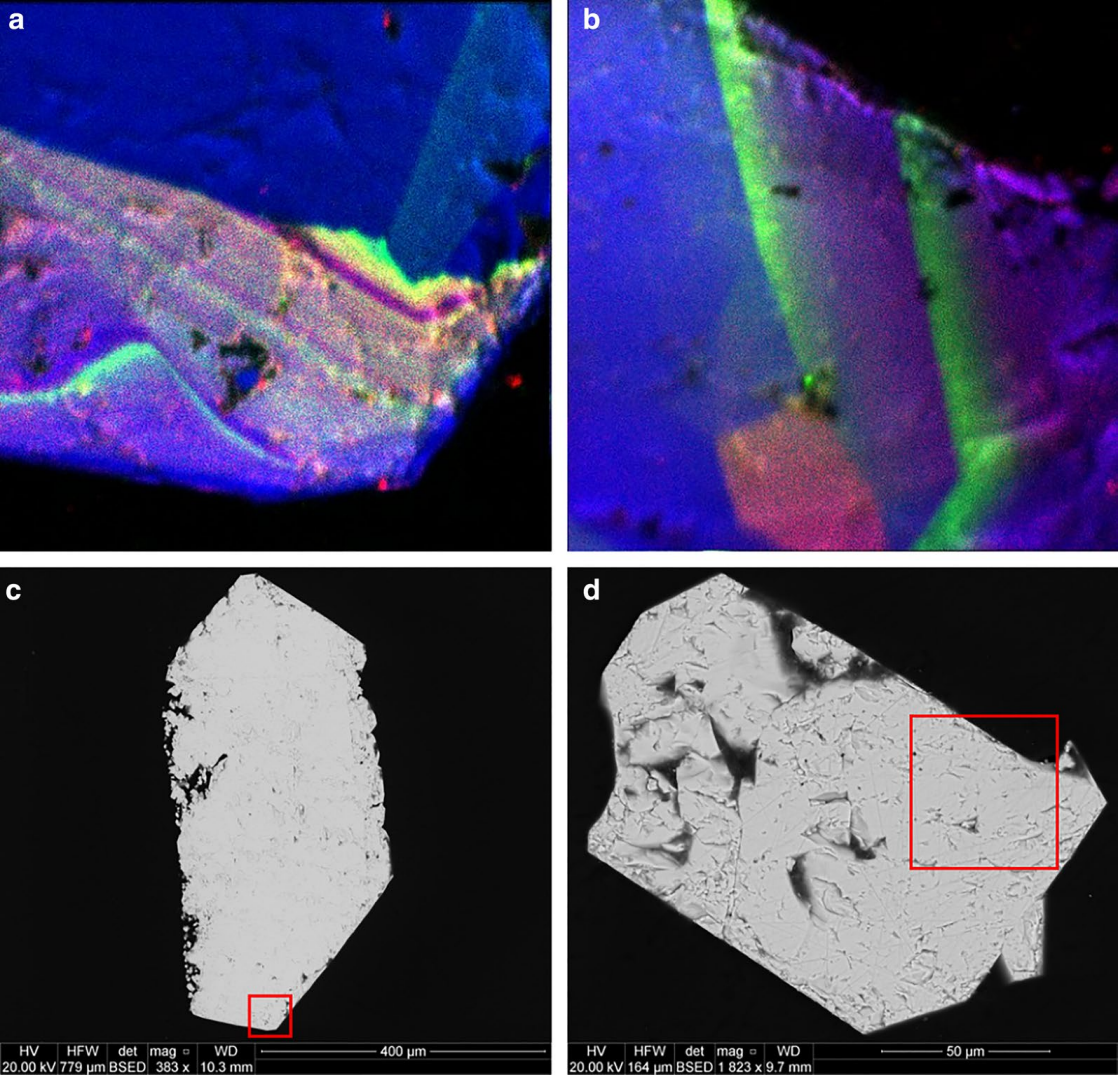
NanoSIMS color composite images (**a**,** b**) and corresponding BSE images (**c**,** d**) of anglesite crystals. Red squares indicate 50 × 50 μm nanoSIMS mapped areas. Diffusion fronts of Ba (green) and Sr (red) can be clearly seen, apparently emulating crystallographic structures of the PbSO_4_ (blue)

Diffusion exists when there is no visible change to the crystal surfaces, although low but measurable concentrations of contaminant elements are present near grain edges. Concentrations tend to decrease with increased depth, as implied in Fig. 15 (experiment 9d). Diffusion may be aided by porosity, as in Fig. 5c, or may be dictated by crystallographic orientation. It is possible, however, for CDR to resemble diffusion, depending on the difference in dissolution and precipitation rates and the amount of resulting porosity. Putnis [26] states “Although volume diffusion is of course always operating, it is doubtful whether it plays a significant role when fluids are present and dissolution–precipitation is another available mechanism”. The key phrase in that statement is “significant role”, although strong evidence exists for a diffusive mechanism. Figure 16 shows nanoSIMS results from a tangential experiment in which diffusion fronts can be seen mimicking crystallographic planes. Concentrations are low, however, and it is unlikely that diffusion (at only 60 °C) was significant in these experiments or will be influential in any future (Ra^2+^, Pb^2+^) removal scenarios.

CDR is a complex mechanism by which a dry-stable compound will undergo dissolution and replacement by an alternatively stable compound when exposed to fluids. Five criteria for identifying CDR have been suggested [27]:

1. Dissolution and precipitation fronts are spatially close. This is crucial for the preservation of morphology of the parent mineral.
2. Sharp reaction fronts dominate, with minimal evidence of solid-state diffusion fronts.
3. The recrystallization product is porous and permeable, allowing continuous fluid contact.
4. Fractures are common ahead of the reaction front, associated with a sufficient increase or decrease in molecular volume.
5. Epitaxial continuity exists across the reaction interface for dissolution/precipitation pairs with similar crystal structures. For dissimilar pairs, a polycrystalline product is observed.

Mineral pseudomorphs are classic examples of this [27, 30]. Figures 12 and 13 show a clear example of anglesite being replaced primarily by (Sr, Pb)SO_4_, with later (Ba, Sr)SO_4_. Data in Fig. 14 suggest temporary stability around Sr:Pb = 3, although the system is clearly not in equilibrium. Reprecipitation lags slightly behind dissolution, with the resultant porosity exceeding 30%—not achievable by molecular volume difference alone. Figures 6b, d and 7b, d confirm these results. With 40 h of low pH SrCl_2_ exposure (Fig. 6b), anglesite shows thick replacement zones on prismatic faces (parallel to b axis) consisting of roughly PbSr(SO_4_)_2_. After 210 h under the same conditions (Fig. 6d), the Sr:Pb dropped to 0.25 but the resulting patchy zones are up to 4 times thicker. Reprecipitation nearly matches dissolution, with porosity of only ~ 10%. Figure 7b, d shows advanced dissolution rims, with Ba-rich PbSO_4_ precipitation lagging. Porosity exceeds 85% at 40 h but is reduced to 70% at 210 h, though the reaction zone has not increased in width. It has been suggested that dissolution and precipitation rates must be necessarily equal to maintain parent crystal morphology [26], yet these timed results suggest there may some room for inequality.

Figure 15 shows the [Sr]/[Pb] for experiments 9(a-d) and [Ba]/[Pb] for experiments 10(a-d). The difference between overgrowth and other uptake methods becomes clear, with thin, nearly pure layers of overgrowth presenting as sharp, symmetric peaks, as seen in the left grain edge of 9c. The right edge of grain 9c is shorter, wider, and asymmetric indicating either diffusion or CDR. Both grain edges of 9d appear to be diffusion but are known to be CDR from textural observations, as are both edges of 10b. The left edge of grain 10d appears to be a hybrid, with a sharp overgrowth peak (or more likely a single particle of BaSO_4_ precipitate adhered to the grain surface) with a much lower concentration of Ba within the grain, decreasing to zero with depth. Naturally, grain orientation in the mount and angle of transect intercept will impart some bias to the shape and amplitude of these peaks, but these ratios can reveal features that are not obvious from transects alone. They emphasize, for example, the preference of Sr uptake over Ba uptake in anglesite.

Similar to diffusion, concentration of contaminants in CDR examples tends to be higher near the original grain surface and decrease with depth. As dissolution progresses, followed by reprecipitation of the new solution components on fresh surfaces, there will be an increase in concentration of the sulfate host cation and a decrease in concentration of the fluid cationic component. As the reaction progresses deeper into the crystal, the solution composition will, to some extent, approach the host end member. This would not apply for well-mixed, endless reservoir systems, but for stagnant limited reservoir systems where diffusion kinetics outperform convection kinetics (as tested here) the effect is evident.

LA-ICP-MS transects show this, with both diffusion fronts and CDR reaction zones recognizable as decreasing product/parent cation ratios towards the center of the crystal (Fig. 15). The relatively large laser spot size, however, results in an averaging effect which swamps finer detail. NanoSIMS images (Figs. 12 and 13) show a general decrease in Ba concentration towards the center of the crystal on a large scale but reveal sharp reaction fronts at high resolution. For this example, four of the five criteria above are met with only Δ_vol_-induced fracturing absent. In this case, fracturing would not be expected as the molecular volume of (Sr_0.75_ Pb_0.25_)SO_4_ is only 2.4% less than the molecular volume of PbSO_4_.

**Fig. 17:**
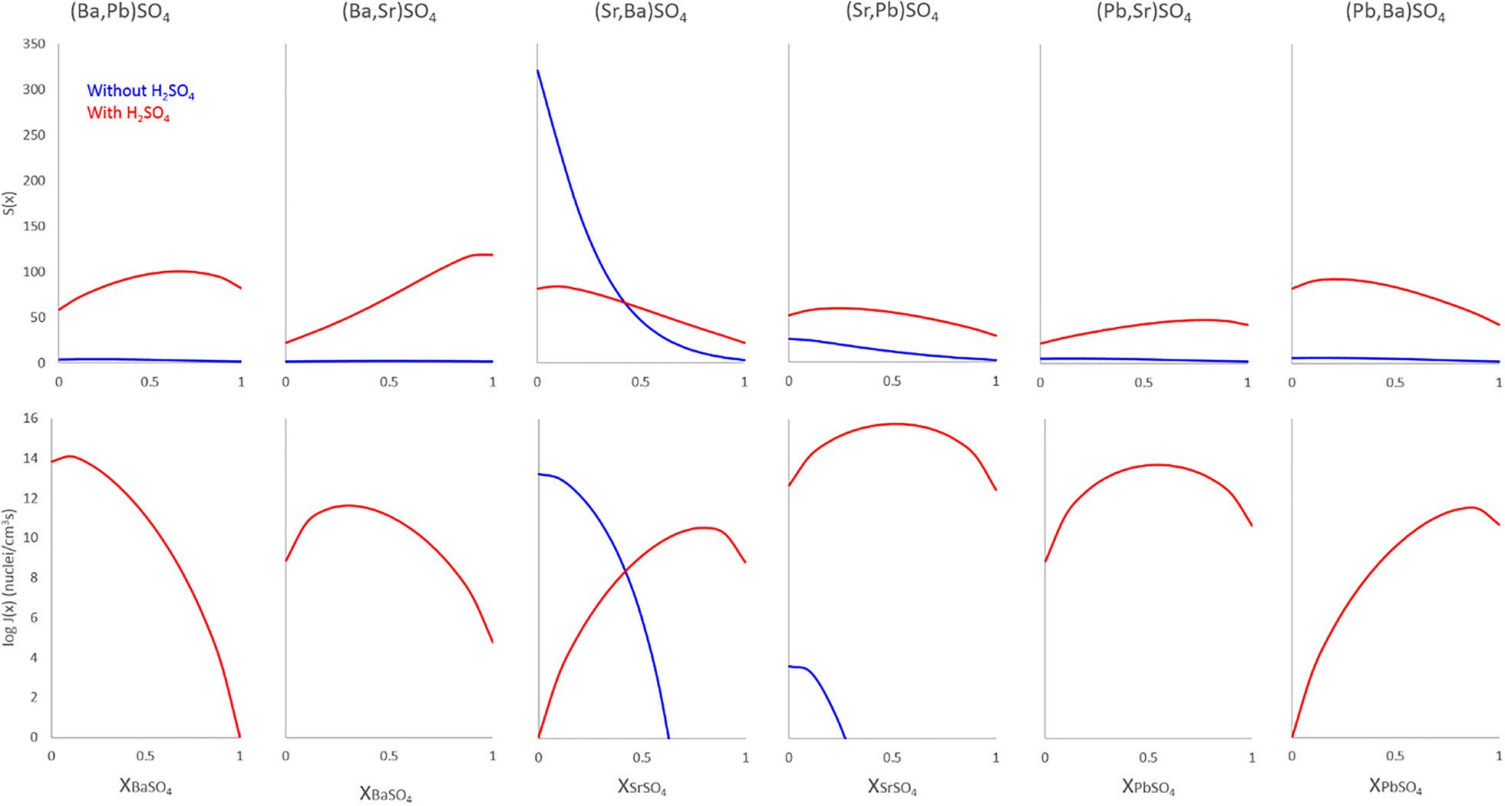
Supersaturation *S(x)* and Nucleation Rate *J*(*x*) models

Porosity is not only a by-product but also a crucial component in CDR [34]. Anglesites in Figs. 6, 7, and 11, 12, 13 show classic CDR textures, similar to those in other published works (Figs. 1a–g and 10a, b in [34]. The rates at which crystals dissolve and reprecipitate (or more accurately, the difference between these rates) will determine not only their new composition, but also the homogeneity and depth of alteration. Based on these rates, minerals may dissolve completely, undergo partial alteration along surfaces and fractures, pseudomorph into an entirely new mineral, or be completely unaffected. Given enough time and preferential metasomatic conditions, mineral grains may be entirely replaced by new minerals with little trace remaining of the original grain other than its shape. Replacement mechanisms are varied, and parent/product compositions may be similar (azurite after malachite, goethite after hematite, etc.), or entirely different (petrified wood, pyrite after aragonite bivalve and gastropod shells; [2]. Judging from the porosity observed in these experiments, replacement of anglesite by celestite (or at least (Sr,Pb)SO_4_) was underway, and was interrupted upon conclusion of the experiment. The same cannot be said for baryte replacement of anglesite, as the porosity seems far too high to sustain complete regrowth. Molecular volume also must be considered, as it is much more difficult to substitute a Ba^2+^ (1.75 Å) into a site normally held by Pb^2+^ (1.63 Å), whereas swapping in a Sr^2+^ (1.58 Å) would be energetically effortless. Porosity would therefore be critical in the incorporation of Ba into PbSO_4_, or Ra (1.84 Å) into any other sulfate, as the increase in molecular volume would necessarily have to be alleviated by pore spaces [34], or substitution pairing with smaller-radii ions (e.g. Mg^2+^, Ca^2+^). Crystal radii from [39]. From starting conditions, it should be possible to predict crystallization products based solely on thermodynamics, but kinetic effects profoundly complicate this. Figure 17 presents the models of all 12 ionic combinations (ignoring the reaction time aspect of the experiments).

### Processing implications

Projecting the above results forward, controlling conditions to maximize replacement mechanisms may be the key for Pb and Ra removal from copper concentrates. Knowing the composition and abundance of non-economic minerals present in the concentrate, and the conditions of acid leaching and subsequent washing, it may be possible to construct a cationic replacement scenario targeting Pb or Ra, or ideally all “insoluble” sulfates.

What sets this study apart from others is that previously, most solubility experiments were performed on isolated compounds. The benefits of this, of course, were simpler data analysis and more accurate data. What was missed, however, was the potential for seeing movement *between* compounds—with no corresponding increase or decrease in overall solubility. With simplistic experiments, it may be assumed that once Sr^2+^, Ba^2+^, or Pb^2+^ ends up in solution, an equilibrium state is met. Once this occurs, the same concentration of ions remains in solution, and is removed during rinsing. During the experiment, liberated ions have nowhere to go other than recrystallizing back on the crystal or remaining in solution. Multiple phases, however, result in multiple potentials which may manifest as mobility between phases (reduction in entropy) with no change in ionic strength of the solution. NanoSIMS images show that there is significant potential for movement between sulfates although solubilities remain low; LA-ICP-MS transects support this hypothesis.

**Fig. 18:**
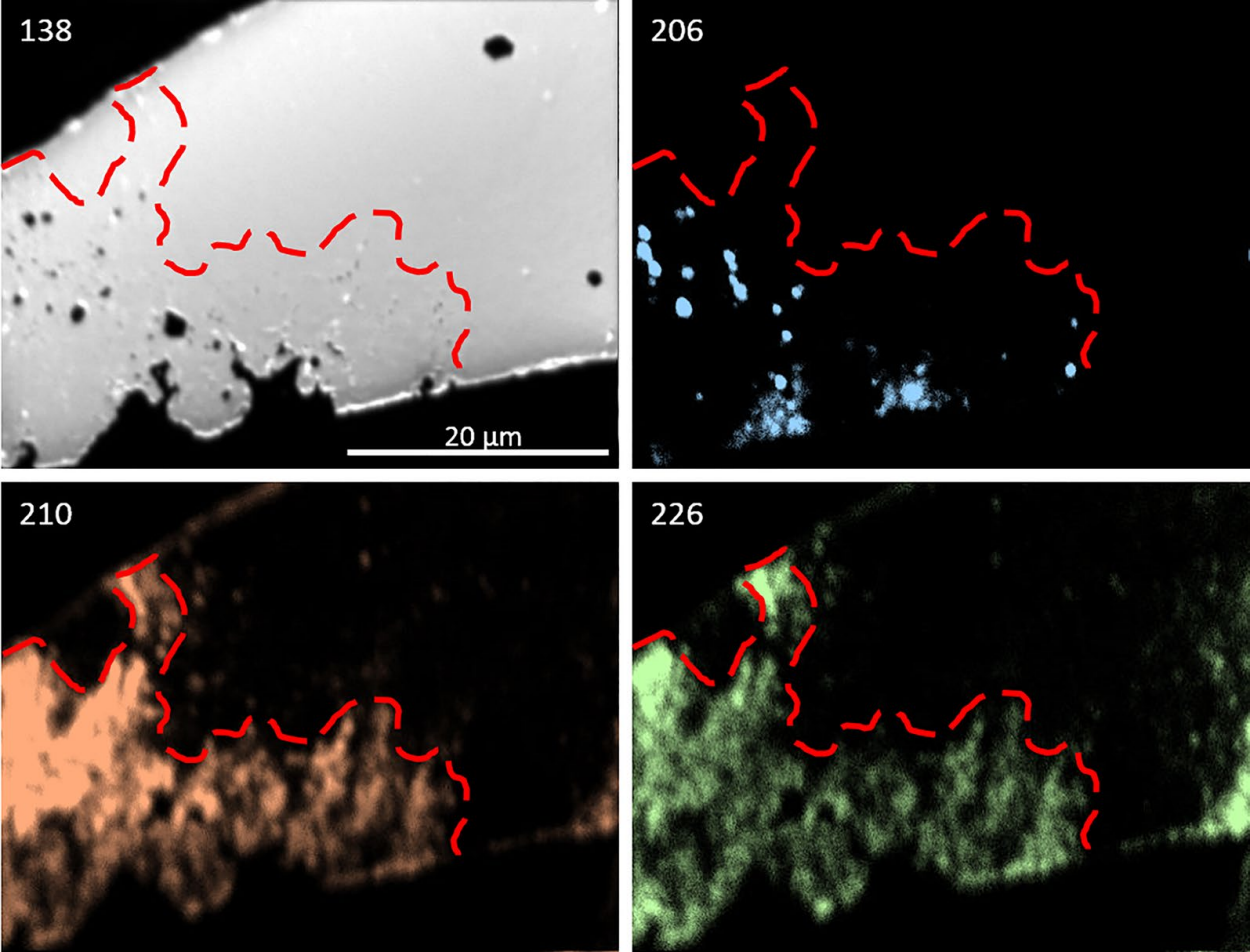
NanoSIMS images of baryte in leached concentrate sample 05CLD 13 from Olympic Dam. The estimated reaction front is marked in red. The non-correlation between ^206^Pb and ^210^Pb indicate multiple pathways; coeval incorporation of old lead into the baryte, uptake of ^210^Pb by the baryte during acid-leaching, and decay of ^210^Pb from ^226^Ra previously taken in by the baryte

Given these results, the question arises: how would they apply to real-world examples? The purpose of this research is to find a practical and efficient pathway for the removal of Ra and lead-210 from copper concentrates. Celestite and anglesite are rare in Olympic Dam ore, although some replacement PbSO_4_ has been found on sulfuric acid-treated galena. Baryte, however, comprises 1.2% of the total orebody [9] and represents the optimal vehicle for an engineered CDR solution. Evidence exists from LA-ICP-MS data of naturally occurring lead uptake in baryte, which increases greatly in the sulfuric acid leach bath [38]. Figure 18 shows nanoSIMS images of baryte exhibiting (CDR-induced?) porosity correlating with ^206^Pb, ^210^Pb, and ^226^Ra concentrations. If CDR is already taking place in the leach tank to some extent, optimizing the process for maximum Pb and Ra removal becomes an achievable goal.

Results from these experiments set the stage for further investigation of radionuclide uptake during minerals processing, especially during acid leaching. At Olympic Dam, the primary RNs of concern during processing are ^226^Ra, ^210^Pb, and ^210^Po. These elements, along with Sr and Ba, represent the entire suite of acid-insoluble sulfates. Considering the commonplace use of sulfuric acid for leaching, understanding the behavior of these sulfates is paramount to controlling their deportment. The evidence provided here enhances our knowledge of this family of micro- to nanoscale chemical interactions and will not only aid in determining where RNs reside during each stage of processing but will also establish the foundation for a plan targeting their removal.

## Conclusions

Results suggest that three distinct mechanisms are involved: overgrowth; diffusion; and CDR. Overgrowth is common for both Pb- and SrSO_4_ on barytes (in low pH, high sulfate solutions), with little evidence that significant amounts of Pb or Sr are incorporated into the baryte structure. Celestite hosts only small amounts of Ba or Pb (generally up to 1 wt%) through porosity-driven diffusion, though very thin overgrowth zones or spots appear sporadically in both cases. Anglesite is readily dissolved and replaced through CDR at low pH conditions, although the effect is somewhat dampened by the presence of excess sulfate. Strontium replacement of Pb averages ~ 75% (m.b.) in acid chloride solution but is reduced to 10 wt% in neutral chloride or acid sulfate solutions. Barium substitution through CDR increases in high-sulfate conditions, but never exceeds 4 wt%. Diffusion of both Ba and Sr into anglesite does occur along crystallographic axes, but only at very low concentrations.

## Data Availability

The data presented above remain the property of, and will only be dispersed at the discretion of, BHP.

## Abbreviations

amu: atomic mass units
BSE: back scatter electron
CDR: coupled dissolution/reprecipitation
CIR: crystal ionic radius
CMCA: Centre for Microscopy, Characterisation, and Analysis
EDS: electron dispersive spectroscopy
FEG: field emission gun
IOCG-U: iron oxide-copper-gold-uranium
LA-ICP-MS: laser ablation inductively coupled plasma mass spectrometry
m.b.: metals basis
nanoSIMS: nanoscale secondary ion mass spectrometry
ppm: parts per million
ppq: parts per quadrillion
ppt: parts per trillion
RN: radionuclide
RO: reverse osmosis
SEM: scanning electron microscopy

## List of symbols

B: geometric factor
J(x): nucleation rate
k: Boltzmann’s constant
K_sp_: solubility product
S(x): supersaturation function
T: temperature (in Kelvin)
Γ(x): preexponential factor
Σ(x): interfacial free energy
Ω(x): molecular volume
